# Updates on CAR T cell therapy in multiple myeloma

**DOI:** 10.1186/s40364-024-00634-5

**Published:** 2024-09-12

**Authors:** Fatemeh Nasiri, Yasaman Asaadi, Farzaneh Mirzadeh, Shahrokh Abdolahi, Sedigheh Molaei, Somayeh Piri Gavgani, Fatemeh Rahbarizadeh

**Affiliations:** 1 Department of Internal Medicine, College of Medicine, Rady Faculty of Health Sciences, University of Manitoba, Winnipeg, MB Canada; 2 Department of Biotechnology, College of Science, University of Tehran, Tehran, Iran; 3 Department of Medical Biotechnology, Faculty of Medical Sciences, Tarbiat Modares University, Tehran, Iran; 4 Basic and Molecular Epidemiology of Gastrointestinal Disorders Research Center, Research Institute for Gastroenterology and Liver Diseases, Shahid Beheshti University of Medical Sciences, Tehran, Iran; 5 School of Medicine, Qom University of Medical Sciences, Qom, Iran; 6 Department of Mycobacteriology and Pulmonary Research, Microbiology Research Center, Pasteur Institute of Iran, Tehran, Iran; 7 Research and Development Center of Biotechnology, Tarbiat Modares University, Tehran, Iran

**Keywords:** CAR T-cell, Multiple myeloma, Antigen heterogeneity, Tumor microenvironment, Toxicities, Combination therapy

## Abstract

Multiple myeloma (MM) is a hematological cancer characterized by the abnormal proliferation of plasma cells. Initial treatments often include immunomodulatory drugs (IMiDs), proteasome inhibitors (PIs), and monoclonal antibodies (mAbs). Despite salient progress in diagnosis and treatment, most MM patients typically have a median life expectancy of only four to five years after starting treatment. In recent developments, the success of chimeric antigen receptor (CAR) T-cells in treating B-cell malignancies exemplifies a new paradigm shift in advanced immunotherapy techniques with promising therapeutic outcomes. Ide-cel and cilta-cel stand as the only two FDA-approved BCMA-targeted CAR T-cells for MM patients, a recognition achieved despite extensive preclinical and clinical research efforts in this domain. Challenges remain regarding certain aspects of CAR T-cell manufacturing and administration processes, including the lack of accessibility and durability due to T-cell characteristics, along with expensive and time-consuming processes limiting health plan coverage. Moreover, MM features, such as tumor antigen heterogeneity, antigen presentation alterations, complex tumor microenvironments, and challenges in CAR-T trafficking, contribute to CAR T-cell exhaustion and subsequent therapy relapse or refractory status. Additionally, the occurrence of adverse events such as cytokine release syndrome, neurotoxicity, and on-target, off-tumor toxicities present obstacles to CAR T-cell therapies. Consequently, ongoing CAR T-cell trials are diligently addressing these challenges and barriers. In this review, we provide an overview of the effectiveness of currently available CAR T-cell treatments for MM, explore the primary resistance mechanisms to these treatments, suggest strategies for improving long-lasting remissions, and investigate the potential for combination therapies involving CAR T-cells.

## Introduction

Multiple myeloma (MM) is still the second most common hematological cancer globally, accounting for about 35,000 diagnoses in the United States and 590,000 worldwide each year, despite years of research and development into better understanding cancer mechanisms and treatment approaches [1, 2]. This aggressive bone marrow (BM) cancer arises from abnormal plasma cells (PCs), which are differentiated B cells [3]. Finding innovative treatments for MM patients who do not respond to standard therapies like IMiDs, PIs, and anti-CD38 monoclonal antibodies is a significant challenge. While the treatment of relapsed or refractory (R/R) MM is evolving to overcome tumor immune evasion mechanisms to improve patient survival, CAR T-cell therapies have shown clinical benefits for blood cancer patients, with six CAR-T products approved by the US FDA since 2017 [4–9]. Notably, Idecabtagene Vicleucel (ide-cel, bb2121, ABECMA) and Ciltacabtagene autoleucel (Cilta-cel, LCAR-B38M, JNJ-68284528, JNJ-4528, CARVYKTI), both approved BCMA-directed CAR-Ts, have demonstrated favorable responses in R/R MM patients. However, despite the prolonged responses of ide-cel and cilta-cel in some patients, a significant number of them eventually face relapse, typically with a response duration of approximately 8.6 months and 34.9 months, respectively [8–13].

This recurrence may be caused by factors such as inherent limitations in T-cell availability and persistence, alterations in T-cell features during treatment, and the nature of the disease, including changes in disease characteristics during treatment [14]. To tackle these issues, CAR-T manufacturing for MM patients has evolved through novel approaches aimed at optimizing and accelerating CAR-T production, reducing costs, and producing cells with a less differentiated phenotypethan conventional CAR T-cells. Additionally, engineering allogeneic T cells as a universal source to express CARs and utilizing CAR expression on other immune cells, such as natural killer (NK) cells, have been explored in various clinical trials [14, 15]. Furthermore, addressing disease features such as variability or shedding of antigen expression on myeloma cells, the presence of an immunosuppressive tumor microenvironment, and difficulties in CAR-T penetration into tumor cells are important issues to improve CAR T-cell efficacy [16, 17]. So, research has explored new designs of CARs, such as adopting dual or multi-specific antigen-targeting receptors, integrating diverse costimulatory domains, and making genetic alterations to co-express cytokines or inhibit intracellular exhaustion signals [18, 19].

Moreover, CAR T-cell treatments often come with undesirable side effects, including cytokine release syndrome (CRS), neurotoxicity, and cytopenia. Approaches to alleviate these effects include combining immunosuppressive drugs or developing innovative direct control systems. Additionally, there are ongoing explorations into creating CAR T-cells utilizing fully humanized monoclonal antibodies (mAbs) to minimize immunogenicity [20, 21].

Consequently, a profound understanding of the therapeutic potential of CAR T-cells, recognition of MM pathogenesis, and development of supplementary therapeutic agents can enhance the effectiveness of transplanted CAR T-cell therapy for MM patients. This article will elucidate various tactics utilized by myeloma cells to counteract the efficacy of CAR-T therapies and delve into advanced approaches to confront these challenges.

## Efficacy and challenges of CAR T-cell therapy in MM clinical trials

CAR T-cell trials for hematological malignancies are more numerous than those for solid tumors [22]. MM is the third most common target for CAR T-cell therapy after B-NHL and ALL [22]. Most clinical trials included both genders, with an average age of 18 and 72, focusing on evaluating anti-tumor efficacy, persistence, and safety profile [22].

**Fig. 1:**
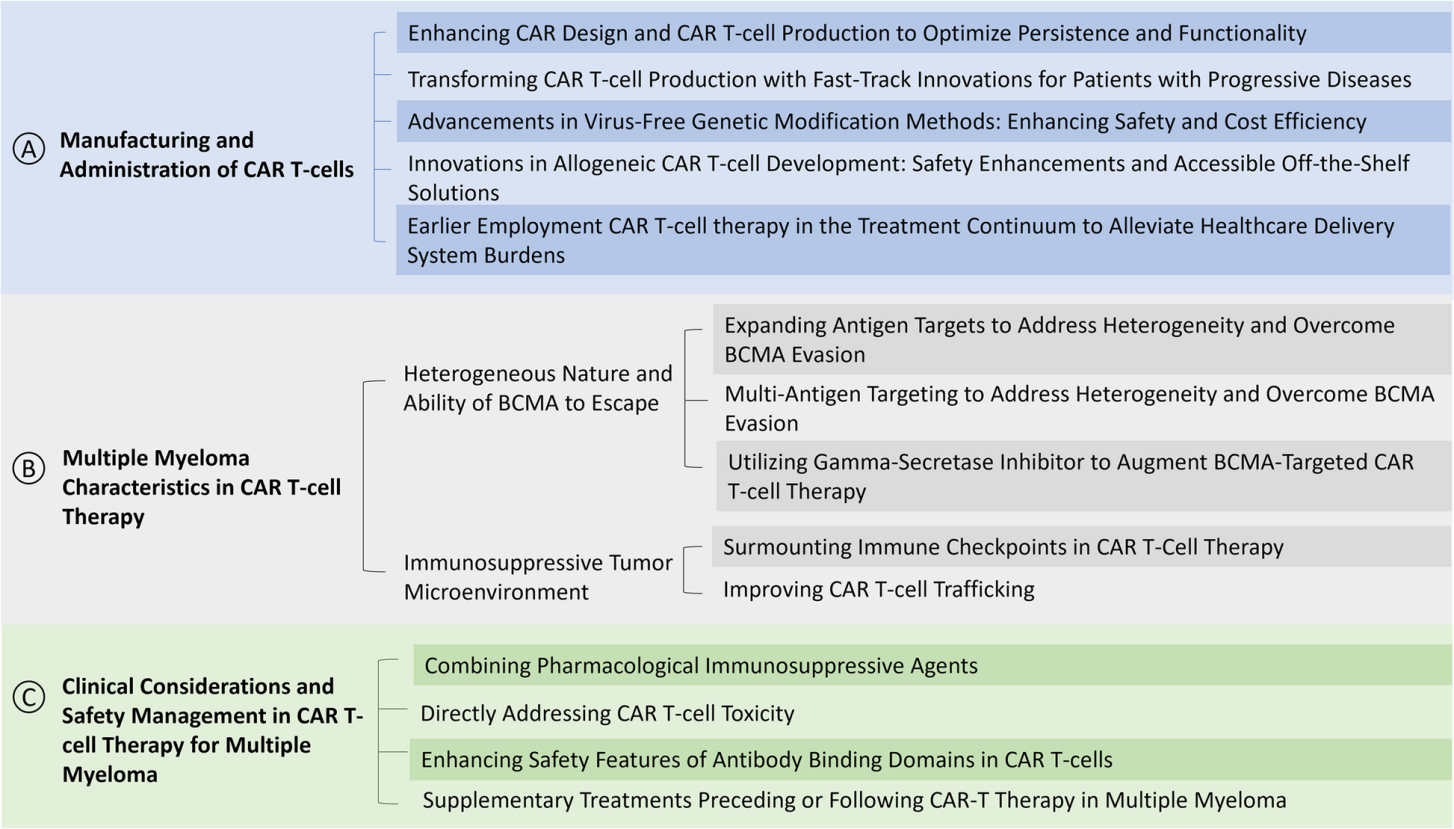
Overview of Effectiveness and Challenges in CAR T-cell Therapy for Multiple Myeloma. **A** Manufacturing and Administration of CAR T-cells: This section highlights strategies for optimizing production and functionality, innovations in genetic modification, allogeneic CAR T-cell development, and early therapy implementation to ease healthcare system burdens. **B** Multiple myeloma Characteristics in CAR T-cell Therapy: It addresses the challenges posed by the heterogeneous nature of the disease and the immunosuppressive tumor microenvironment. Solutions include expanding antigen targets, using gamma-secretase inhibitors, surmounting immune checkpoints, and improving CAR T-cell trafficking. **C** Clinical Considerations and Safety Management: This part emphasizes the importance of managing CAR T-cell toxicity and enhancing safety features. It suggests combining pharmacological agents and supplementary treatments to improve overall therapy outcomes

Several factors affect the effectiveness of CAR T-cell therapy for MM, which can be classified into three main categories: CAR T-cell manufacturing and administration processes, disease characteristics, and clinical considerations before or after CAR T-cell treatment (Fig. 1). The subsequent sections investigate in extensive detail the clinical trials conducted thus far on CAR T-cell therapy in the realm of MM disease.

## Manufacturing and administration of CAR T-cells

### Autologous CAR T-cell manufacturing

The standard process of making autologous CAR T-cells entails activating T cells from patients, genetically modifying them with CARs using retroviruses or lentiviruses, and expanding these cells ex vivo for at least two weeks before reintroducing them into the patient [23, 24]. CARs have an extracellular single-chain variable fragment (ScFv) for tumor antigen recognition without relying on major histocompatibility complex (MHC) restricted antigen presentation. Most CAR constructs incorporate at least one intracytoplasmic costimulatory domain, often called “second-generation,” such as CD28, 4-1BB, ICOS, or OX40, along with CD3ζ. Specific CAR designs incorporate additional costimulatory domains, cytokine receptors, or safety switches into the CAR structure, commonly termed “third- or fourth-generation CARs [25, 26]. Ide-cel and cilta-cel are both second-generation autologous CAR T-cell products. They employ the 4-1BB costimulatory domain, which has demonstrated effectiveness in the proliferation and persistence of CAR-Ts compared to other options. Both products utilize a third-generation lentiviral vector for CAR gene transfer and follow a similar approach to CAR T-cell expansion [9, 10].

### Enhancing CAR design and CAR T-cell production to optimize persistence and functionality

During the initial phases of CAR T-cell therapy development, there was a belief that the sheer quantity of CAR T-cells administered would be a crucial factor in the therapy’s effectiveness. However, it has become apparent that beyond a certain point, the absolute number of infused CAR-Ts doesn’t directly correspond to their persistence and therapeutic success [27]. Consequently, researchers are exploring new methods in CAR T-cell production and administration to improve their longevity and efficacy.

In the case of MM patients, many are older and have lower T-cell counts and an altered CD4/CD8 ratio before undergoing CAR T-cell therapy, which can affect their T cells’ fitness. To address this issue, several clinical trials adjusted the CD4/CD8 ratio to 1:1 in the case of BCMA-directed CAR-Ts, such as orvacabtagene autoleucel (Orva-cel; JCARH125) (NCT03430011), or after gene transfer in the case of FCARH143 (NCT03338972) [28, 29] (Table 1).

**Table 1:** Clinical trials addressing challenges in CAR-T cell manufacturing and administration in multiple myeloma

					Adverse effect	Outcomes			
Target/CAR-T Name	Strategy/Source	Phase/Study Name	Status	No. of Patient	Grade ≥3 CRS/ICANS%	ORR%	Median PFS, months	Location	NCT
BCMA (Orva-cel; JCARH125)	CD4/CD8	I/II (EVOLVE)	Completed	165	2/4	91	NA	US	NCT03430011 [29]
BCMA (FCARH143)	CD4/CD8	I	Completed	21	0/0	100	NA	US	NCT03338972 [28]
BCMA (Descartes-08)	CD8+, mRNA	I/II	Completed	67	NA	NA	NA	US	NCT03448978
BCMA (Descartes-08)	Earlier employment, CD8+, mRNA	II	Terminated	30	NA	NA	NA	US	NCT04816526
BCMA (Descartes-11)	CD8+, mRNA	I/II	Not recruiting	25	0/0	NA	NA	US	NCT03994705
BCMA (Descartes-11)	Earlier employment, CD8+, mRNA	II	Completed	30	NA	NA	NA	US	NCT04436029
BCMA (Bb21217)	Memory-like T, PI3K inhibitor	I (CRB-402)	Completed	72	4/7	55	11.9	US	NCT03274219 [30]
BCMA (ide-cel)	Earlier employment	I (KarMMa-4)	Completed	13	NA	NA	NA	US	NCT04196491
NKG2D (CYAD-01)	Earlier employment	I/II (THINK)	Unknown	146	31/NA	NA	NA	US	NCT03018405 [31]
BCMA/CD19 (GC012F)	FasTCAR	I/II	Recruiting	20	0/0	100	Not yet reached	China	NCT04935580 [32]
BCMA/CD19 (GC012F)	FasTCAR	Early I	Unknown	18	2/0	93.1	38	China	NCT04182581 [33]
BCMA/CD19 (GC012F)	FasTCAR	Early I	Unknown	15	2/0	93.1	38	China	NCT04236011 [33]
BCMA/CD19 (GC012F)	FasTCAR	Early I	Unknown	15	NA	NA	NA	China	NCT04617704
BCMA/CD19 (GC012F)	FasTCAR	I/II	Recruiting	68	NA	NA	NA	US	NCT05850234
BCMA (PHE885)	T-Charge TM	I	Not recruiting	96	11/7	98	Not yet reached	US	NCT04318327 [34]
BCMA (CC-98633)	NEX-T CAR	I	Not recruiting	150	1.5/0	95.1	NA	US	NCT04394650 [35]
BCMA (P-BCMA-101)	Transposon, Tscm	I/II	Terminated	105	2/0	57	NA	US	NCT03288493 [36]
BCMA (P-BCMA-101)	Transposon, Tscm	I	Not recruiting	100	NA	NA	NA	US	NCT03741127
BCMA (P-BCMA-ALLO1)	Transposon, Tscm, Cas-CLOVER, Allogeneic	I (MM)	Recruiting	135	0/0	82	Not yet reached	US	NCT04960579 [37]
BCMA (BCMA-UCART)	CRISPR/Cas9, Allogeneic	Early I	Suspended	20	2/0	95	Not yet reached	China	NCT03752541
BCMA (PBCAR269A)	ARCUS, Allogeneic	I	Terminated	48	NA	NA	NA	US	NCT04171843
BCMA (ALLO-605)	TALEN, Allogeneic	I/II (IGNITE)	Not recruiting	136	NA	NA	NA	US	NCT05000450
BCMA (ALLO-715)	TALEN, Allogeneic	I (UNIVERSAL)	Not recruiting	132	2/0	61.5	Not yet reached	US	NCT04093596 [38]
BCMA (CTX120)	CRISPR/Cas9, Allogeneic	I	Not recruiting	26	NA	NA	NA	US	NCT04244656
BCMA (CB-011)	CRISPR-Cas12a, Allogeneic	I (CaMMouflage)	Recruiting	50	NA	NA	NA	US	NCT05722418
BCMA (CYAD-211)	shRNA/Hairpin, Allogeneic	I (IMMUNICY-1)	Not recruiting	18	0/0	NA	NA	US	NCT04613557 [39]
SLAMF7 (UCARTCS1A)	TALEN, Allogeneic	I (MELANI-01)	Terminated	11	NA	NA	NA	US	NCT04142619
BCMA (CAR-NK 92)	NK-92 cell	I/II	Unknown	20	NA	NA	NA	China	NCT03940833
BCMA	Allogeneic NK	Early I	Recruiting	19	NA	NA	NA	China	NCT05652530
BCMA	CB-NK	Early I	Unknown	27	NA	NA	NA	China	NCT05008536
BCMA	NK	Early I	Recruiting	18	NA	NA	NA	China	NCT06045091
BCMA (FT576)	NK, iPSCs	I	Recruiting	168	NA	NA	NA	US	NCT05182073

B-cell maturation antigen (BCMA), Cluster of differentiation (CD), Signaling lymphocyte activation molecule family-7 (SLAMF-7), Natural-killer group 2 member D (NKG2D), T stem cell memory (Tscm), Clustered regularly interspaced short palindromic repeat (CRISPR)-associated protein 9 (CRISPR/Cas9), Short/small hairpin RNA (shRNA/Hairpin), Transcription activator-Llike effector nucleases (TALENs), natural killer (NK) cells, Induced pluripotent stem cells (iPSCs), Cord blood-derived natural killer cells (CB-NK), Cytokine release syndrome (CRS), Immune effector cell-associated neurotoxicity syndrome (ICANS), Overall response rate (ORR), and Progression-free survival (PFS)

Furthermore, a significant limitation of CAR T-cell therapy stems from the restricted lifespan of the transplanted T cells, a characteristic mainly linked to their inherent properties and the absence of memory attributes [24]. Current research indicates that augmenting CAR T-cell cultures with recombinant interleukins such as IL-5, IL-7, IL-8, IL-12, IL-15, IL-21, IL-23, and IL-36γ can enhance their persistence and promote a higher ratio of memory cells compared to effector cells [40]. In a preliminary clinical trial, a fourth-generation CAR T-cell targeting BCMA, which expresses IL-7 and CCL19 (known as 7 × 19 CAR T-cells), demonstrated superiority over traditional second-generation CAR T-cells in terms of expansion, accumulation of memory cells, migration, and cytotoxicity (NCT03778346) [41].

Recently, a phase I open-label trial (NCT04136756) investigated NKTR-255 in patients with R/R MM who had progressed after prior CAR-T therapy. NKTR-255 is an experimental IL-15 receptor agonist designed to boost Natural Killer (NK) cells and memory CD8 + T cells [42]. Accordingly, some clinical trials, including anti-BCMA CAR-Ts like Descartes-08 (NCT03448978, NCT04816526) and Descartes-11 (NCT03994705, NCT04436029), employed CD8 + cells, while P-BCMA-101 (NCT03288493, NCT03741127) and P-BCMA-ALLO1 (NCT04960579) utilized the T stem cell memory (Tscm) phenotype in their approaches [36, 37, 43]. Bb21217 also used the same CAR construct as ide-cel (bb2121); however, a PI3K inhibitor (bb007) was added to the ex vivo culturing process to enhance the memory-like T cell subpopulations (NCT03274219) [44]. The clinical pursuit of bb21217 showed an overall response rate (ORR) of 55%, which led to the completion of its development [30] (Table 1).

### Transforming CAR T-cell production with fast-track innovations for patients with progressive diseases

The duration required for CAR T-cell production and the type of protocol used in ex vivo settings directly impact T cells’ vital outcomes such as cytotoxicity, cytokine production against target cells, persistence, and the prevalence of more resilient phenotypes like naïve, memory, and stem cell memory in T cells [23]. Leveraging the unique ability of lentiviral vectors to integrate into non-dividing cells, researchers have developed a highly efficient method to accelerate the production of CAR T-cells. This groundbreaking approach has slashed the manufacturing timeline from weeks to just one to two days, resulting in rapid CAR T-cell production with improved growth and reduced exhaustion [45, 46].

GC012F, a dual-targeting CAR-T product for BCMA/CD19 enabled by FasTCAR technology, is currently undergoing investigation in a Phase I trial conducted across multiple centers for MM in China (NCT04236011, NCT04182581, NCT04617704, NCT04935580, ChiCTR2100047061) [47]. Phase Ib/II trials for GC012F are also underway in the US and China (NCT05850234) [48, 49].

Additionally, CC-98633/BMS-986354, an enhanced version of orva-cel, a lentiviral CAR T-cell product that targets BCMA, employs the NEX-T approach to shorten the manufacturing period to 5 to 6 days. It is presently under evaluation in an ongoing phase I clinical trial (NCT04394650). Clinical studies have demonstrated that BMS-986354 is a less mature CAR T-cell product with enhanced effectiveness compared to Orva-cel, making CAR T-cell therapy more accessible and suitable for patients with progressive diseases [35].

PHE885, a fully human BCMA CAR T-cell product, is also rapidly manufactured with the T-Charge™ platform (< 2 days). It is currently undergoing Phase I trial evaluation (NCT04318327) to assess CAR T-cell expansion within the patient’s body [34, 50] (Table 1).

### Advancements in virus-free genetic modification methods: enhancing safety and cost efficiency

Due to the complexity, extended duration, and high costs associated with viral vectors, there is a concerted effort to explore virus-free genetic modification methods in CAR T-cell manufacturing. These methods include transposon systems like PiggyBac (PB) and Sleeping Beauty (SB), as well as zinc-finger nucleases (ZFNs), transcription activator-like effector nucleases (TALENs), short/small hairpin RNA (shRNA/Hairpin) vectors, and clustered regularly interspaced short palindromic repeat (CRISPR)-associated protein 9 (CRISPR/Cas9). Additional approaches include ARCUS, a specific variant of the CRISPR/Cas9 gene editing system that serves as a fully synthetic enzyme derived from the natural homing endonuclease I-CreI, and Cas-CLOVER, which utilizes a dual-guided system to create double-strand cuts upon dimerization with nuclease Clo051. All of these methods have demonstrated gene edition efficiency in CAR T-cell manufacturing [51, 52].

In the pursuit of enhancing CAR T-cell production quality, a novel category of CAR-Ts known as Descartes-08, derived from anti-BCMA mRNA, has been developed to reduce excessive in vitro proliferation time. The therapy has been evaluated in two clinical trials, identified as NCT03448978 and NCT04816526. The former has completed a phase I/II trial, while the latter has terminated a recent phase II trial.. Further, Descartes-11, an optimized and humanized version of Descartes-08, is under clinical trials (NCT03994705, NCT04436029) [43].

Additionally, P-BCMA-101 is a fully humanized anti-BCMA CAR T-cell employing a novel anti-BCMA VCAR with a non-immunoglobulin Centyrin scaffold produced using the transposon system. These VCARs, smaller than immunoglobulin-based CARs, offer advantages such as enhanced binding affinity, improved stability, reduced immunogenicity, and cost-effective production. P-BCMA-101 terminated a phase I trial (NCT03288493) in heavily treated R/R MM patients, achieving a 57% ORR [36]. A 15-year follow-up study (NCT03741127) has been initiated to delve deeper into the product’s efficacy and safety. Leveraging P-BCMA-101 data, an allogeneic CAR-T, P-BCMA-ALLO, using a similar PB construct was created and demonstrated excellent clinical safety and effectiveness in a phase I trial (NCT04960579) [36, 37, 52] (Table 1).

### Innovations in allogeneic CAR T-cell development: safety enhancements and accessible off-the-shelf solutions

A group of ongoing research is dedicated to developing allogeneic CAR T-cell products that can be readily accessible “off the shelf” to patients with relapsed or refractory conditions. Such patients often confront rapid disease progression during the production of CAR-Ts or have diminished T-cell counts, rendering the receipt of autologous CAR T-cell therapy both time-consuming and financially burdensome. These limitations may result in missed opportunities for CAR T-cell treatment. To mitigate the risk of graft-versus-host disease (GvHD) and to enhance the cost-effective and expedited worldwide production and prescription procedures, cutting-edge genetic editing techniques are employed. These techniques either deactivate specific genes, such as the T cell receptor (TCR) αβ or γδ chains and MHC in T cells derived from healthy, universal donors, or introduce specific genes like CARs [51–53].

UCARTCS1A, the pioneering allogeneic CAR-T cell therapy in clinical development for MM, has eliminated both the SLAMF-7 antigen and TCR from T-cell surfaces by utilizing TALEN technology. This process also involved the integration of the SLAMF-7 CAR construct, and phase I trials for this therapy have been terminated (NCT04142619) [54]. Other treatments, ALLO-715 (NCT04093596) and ALLO-605 (NCT05000450), which are modified allogeneic anti-BCMA CAR T-cells, use TALEN technology to deactivate the T-cell receptor alpha constant gene (TRAC) and CD52. They are administered with ALLO-647, an anti-CD52 mAb, allowing for selective and temporary lymphodepletion before CAR-T therapy [51, 55, 56].

Moreover, P-BCMA-ALLO1 utilized Cas-CLOVER to disrupt β2 microglobulin (B2M), a component of MHC-I, as well as the TCRβ chain in T cells (NCT04960579) [37, 52]. In a terminated trial, PBCAR269A employed ARCUS technology to introduce TCR knock-outs and CAR T knock-ins for allogeneic BCMA-CAR T cells (NCT04171843).

BCMA-UCART utilized CRISPR/Cas9 (NCT03752541), while CB-011 employs CRISPR-Cas12a (NCT05722418) to target TRAC and MHC-I [57, 58]. In a similar vein, CTX120 is generated using the CRISPR/Cas9 system to remove TCR and MHC-I, followed by the specific insertion of the CAR at the TRAC locus using an AAV vector (NCT04244656) [59]. Furthermore, CYAD-211 is developed using shRNA/Hairpin and is currently under evaluation in the ongoing Phase I exploratory trial IMMUNICY-1 (NCT04613557) [39, 52] (Table 1).

Besides, double-negative T cells (DNT) are mature T cells that lack the CD4 and CD8 co-receptors on their surface. DNTs, recognized for their suppressive capabilities and non-specific antigen recognition, have demonstrated the ability to suppress GVHD independent of MHC limitations, indicating their potential role in allogeneic transplantation scenarios [60]. These cells have been studied in the context of allogeneic transplantation and cancer therapy, and they have been shown to persist in recipients for at least four weeks alongside conventional T cells without eliciting cytotoxic responses [61, 62]. Allogeneic double-negative CAR-T cells have demonstrated robust efficacy in inhibiting tumor growth in mouse models of blood cancers, suggesting their potential as a novel source for “off-the-shelf” CAR-T cell therapy [60].

Natural killer (NK) cells also possess inherent properties that make them suitable candidates for allogeneic cell therapy applications. Their ability to recognize and eliminate stressed or transformed cells without prior sensitization, coupled with a reduced risk of GVHD, positions them as promising targets for developing off-the-shelf allogeneic immunotherapies [63]. Several clinical trials are currently underway to evaluate BCMA-targeted CAR-NK cells for the treatment of multiple myeloma, such as NCT05652530, NCT05008536, NCT03940833, NCT06045091, and NCT05182073 [64, 65] (Table 1).

### Earlier employment CAR T-cell therapy in the treatment continuum to alleviate healthcare delivery system burden

In the most recent clinical trials, CAR T-cell therapy is typically administered after multiple rounds of prior treatments for patients with R/R MM. However, the efficacy of successive therapies tends to diminish, with decreasing response rate, shallower depth of response, and shorter times to disease progression. Several factors contribute to this decline, including the development of resistance mechanisms, accumulation of treatment-related toxicities, selection of treatment-resistant cancer cell populations, and compromised T cell function due to prior therapies [23]. These phenomena pose challenges for implementing CAR-T treatment in later stages.

Cilta-Cel and Ide-cel are approved for myeloma patients undergoing disease progression after standard treatment with PIs, IMiDs, and anti-CD38 mAb. These approvals are based on the requirement that patients must have been exposed to triple-class therapy and received three or four prior lines of treatment in the EU and US, respectively [66]. On April 4, 2024, a significant modification to the approved Risk Evaluation and Mitigation Strategy (REMS) for Ide-cel was approved to reduce the burden on the healthcare delivery system. This modification applies to the treatment of adult patients with RR/MM who have undergone at least two prior therapies.

Additionally, the Phase I (THINK) study (NCT03018405) is currently evaluating the effectiveness of CYAD-01 without prior chemotherapy or lymphodepletion in MM patients, aiming for earlier employment of CAR T-cells [31, 67]. Similarly, phase II clinical trials for Descartes-08 (NCT04816526) and Descartes-11 (NCT04436029), along with completed safety assessments in the KarMMa-4 trial for ide-cel (NCT04196491), have been undertaken to evaluate the effectiveness of these interventions for individuals with newly diagnosed or high-risk multiple myeloma [65] (Table 1).

## Multiple myeloma characteristics in CAR T-cell therapy

### Heterogeneous nature and ability of BCMA to escape

Some particular antigens expressed on plasma cells can efficiently recruit specific CAR-Ts for MM. BCMA (CD269/TNFRSF17), with near-universal expression in MM, is the most intensively studied target for MM CAR T-cell treatments. One of the barriers to the success of CAR-T therapy is antigen heterogeneity, which interferes with T lymphocyte recognition and reduces the efficacy of the treatment [68]. The variable distribution of BCMA on myeloma cells and BCMA’s ability to escape immune cells is associated with resistance to current anti-BCMA CAR T-cell therapies. However, the underlying mechanisms of this phenomenon are still poorly understood [69].

**Fig. 2:**
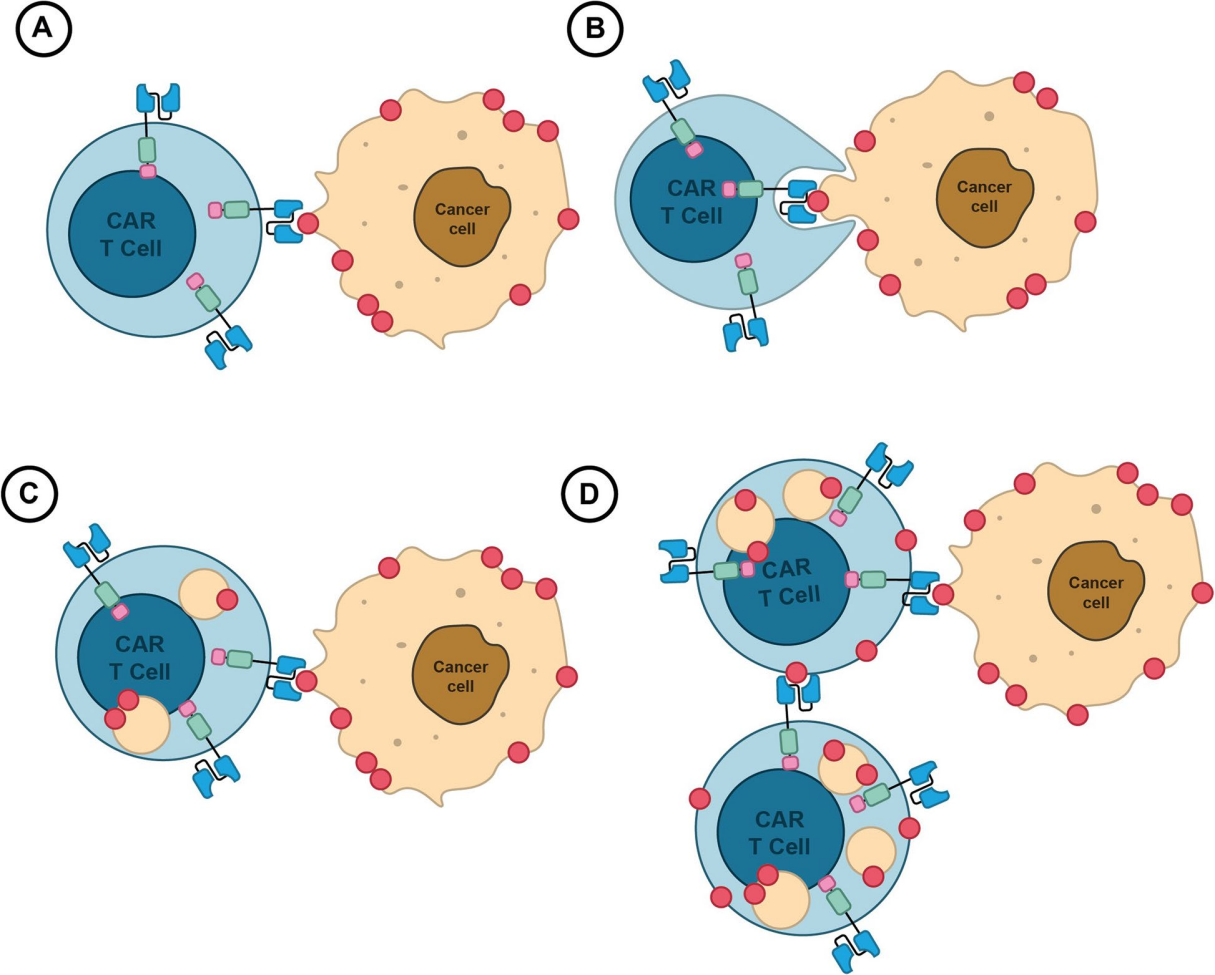
BCMA Transfer Mechanism in CAR T Cell Therapy. **A** Direct Contact: CAR T cells engineered to target BCMA form an immunological synapse with tumor cells, facilitating molecular exchange. **B** Trogocytosis: Membrane fragments containing BCMA are transferred from tumor cells to CAR T cells during synapse formation via trogocytosis, enhancing T cell activation. **C** BCMA Recognition: Transferred BCMA molecules are recognized by CARs on T cell surfaces, initiating T cell activation and tumor cell destruction. **D** Implications: While BCMA transfer boosts T cell killing, trogocytosis may inadvertently lead to fratricide, reducing therapy efficacy

Inherently, BCMA cleaved by gamma-secretase (a membrane-bound protease) can be released into the circulatory system or transferred spontaneously from tumors to T cells by a process known as trogocytosis, which causes T-cell fratricide (self-cytotoxicity) [70] (Fig. 2). This phenomenon may increase the risk of disease recurrence by making it more difficult for anti-BCMA CAR-Ts to find malignant cells [68, 71]. Consequently, the BCMA expression thresholds are currently being debated, and various results have been published regarding the use of BCMA expression thresholds as inclusion criteria for anti-BCMA CAR trials (NCT02546167, NCT03338972) [70, 72]

Furthermore, one primary process of MM recurrence and refractoriness is the reduction of antigens or their disappearance under therapeutic pressure. While analyzing ide-cel, researchers discovered new resistance mechanisms of BCMA escape. They observed the downregulation of BCMA through non-truncating missense mutations or in-frame deletions in the extracellular domain of BCMA, in addition to tumor intrinsic resistance mechanisms that occurred at relapse after anti-BCMA CAR T therapy [73]. While both alleles were present before CAR T therapy, the majority of the MM cells displayed heterozygous deletion of the BCMA locus-containing region of chr16p together with nonsense mutation of the other allele, resulting in a biallelic suppression of BCMA [74]. So, defining tumor phenotypes before and after treatment may correlate with different clinical observations in the future [73]. Upon progression, BCMA loss might play a role in preventing the reapplication of the same CAR-Ts. Accordingly, cilta-cel’s unique structural design, with two tandemly arranged BCMA-targeting VHH (Variable heavy chain of heavy chain only antibody found in camelids) domains recognizing two different epitopes of antigens, exhibited a considerable therapeutic impact in R/R MM patients [10, 75, 76].

In the pivotal phase 2 KarMMa trial (NCT03361748), participants who received ide-cel infusion demonstrated an ORR of 73%, a median PFS of 8.6 months, and a median overall survival (OS) of 24.8 months [8, 77]. Meanwhile, the phase I/II CARTITUDE-1 trial (NCT03548207) demonstrated that cilta-cel delivered an impressive 98% ORR with a median PFS of 34.9 months [8, 11, 78]. In phase 3 trials, specifically CARTITUDE-4 (NCT04181827) and KarMMa-3 (NCT03651128), updated data was used to evaluate ide-cel and cilta-cel with a control arm, although these trials had different designs and patient populations. The results once again confirmed the effectiveness of ide-cel and cilta-cel treatments, with a hazard ratio for PFS of 0.49 for ide-cel [95% CI, 0.38–0.65; *P* < 0.001], and 0.26 for cilta-cel [95% CI, 0.18–0.38; *P* < 0.001] [12, 13, 79].

#### Expanding antigen targets to address heterogeneity and overcome BCMA evasion

Due to the limitations associated with targeting BCMA, numerous clinical trials are currently underway to investigate the incorporation of various intriguing antigens into CART cell therapy in MM therapy. These include some antigens such as signaling lymphocyte activation molecule family-7 (SLAMF-7) (expression in 95% MM), CD138 (expression in 90–100% MM), CD38 (expression in 80–100% MM), CD56 (expression in 70–90% MM), Kappa (κ) light chain (expression in 35% MM) as well as overexpressed antigens like natural-killer group 2 member D (NKG2D), G protein-coupled receptor class C group 5 member D (GPRC5D), integrin‑Beta7, MMG49, CD44v6, CD74, NY-ESO-1, TnMUC1, APRIL, TACI and CD19 [71, 80] (Table 2).

**Table 2:** Clinical Trials Addressing Limited Efficacy of BCMA CAR-Ts Due to Multiple Myeloma Characteristics

					Adverse effects	Outcomes			
Strategies Under Investigation	Target/CAR-T Name	Phase/Study Name	Status	No. of Patient	Grade ≥ 3 CRS/ICANS,%	ORR,%	Median PFS, months	Location	NCT
Using tandem or multiplexed BCMA CAR-T cell therapies to address BCMA reduction or evasion	BCMA/CD19	I	Unknown	20	NA	NA	NA	China	NCT04194931
	BCMA/CD19	Early I	Recruiting	120	NA	NA	NA	China	NCT04603872
	BCMA/CD19	Early I	Unknown	18	NA	NA	NA	China	NCT04412889
	BCMA/CD19	I (MCARTY)	Not recruiting	24	NA	100	Not yet reached	UK	NCT04795882 [81]
	BCMA/CD19 (GC012F)	I/II	Recruiting	20	0/0	100	Not yet reached	China	NCT04935580 [32]
	BCMA/CD19 (GC012F)	Early I	Unknown	18	2/0	93.1	38	China	NCT04182581 [33]
	BCMA/CD19 (GC012F)	Early I	Unknown	15	2/0	93.1	38	China	NCT04236011 [33]
	BCMA/CD19 (GC012F)	Early I	Unknown	15	NA	NA	NA	China	NCT04617704
	BCMA/CD19 (GC012F)	I/II	Recruiting	68	NA	NA	NA	US	NCT05850234
	BCMA/CD19	I	Unknown	20	NA	NA	NA	China	NCT03706547
	BCMA/CD19 (Cocktail)	I/II	Recruiting	15	0/0	100	NA	China	NCT03455972 [82]
	BCMA/CD19	I	Recruiting	12	2/NA	95	8	China	NCT04162353 [83]
	BCMA/CD19	I	Not Recruiting	40	NA	NA	NA	USA	NCT03549442
	BCMA/CD19	I/II	Recruiting	24	NA	NA	NA	China	NCT04714827
	BCMA/SLAMF-7	I	Recruiting	24	6/0	81	9	China	NCT04662099 [84]
	BCMA/SLAMF-7	Early I	Unknown	12	NA	NA	NA	China	NCT04156269
	BCMA/GPRC5D (MCARH125/MCARH109)	I	Recruiting	24	NA	NA	NA	US	NCT05431608
	BCMA/GPRC5D	NA	Recruiting	40	NA	NA	NA	China	NCT06068400
	BCMA/GPRC5D	I	Not yet recruiting	9	NA	NA	NA	China	NCT05325801
	BCMA/GPRC5D	II	Recruiting	10	NA	NA	NA	China	NCT05998928
	BCMA/GPRC5D	II	Recruiting	30	NA	NA	NA	China	NCT05509530
	BCMA/CD38	I/II	Recruiting	80	NA	NA	NA	China	NCT03767751
	BCMA/TACI (AUTO2)	I/II	Terminated	12	0/0	45.5	NA	UK	NCT03287804 [85]
	BCMA/TACI (APRIL)	Early I	Recruiting	36	NA	NA	NA	China	NCT04657861
	BCMA/TACI (TriPRIL)	I	Recruiting	18	NA	NA	NA	US	NCT05020444
	BCMA, CD19, CD138, more	I/II	Recruiting	10	0/0	90	Not yet reached	China	NCT03196414 [86]
	BCMA, CD38, CD138, CD56	NA	Terminated	2	NA	NA	NA	China	NCT03473496
	BCMA, CD38, CD138, CD56	I/II	Unknown	20	NA	NA	NA	China	NCT03271632
	BCMA, CD38, CD138, SLAMF-7, Integrin β7	I	Recruiting	18	NA	NA	NA	China	NCT03778346
	CD19 and CD20/CD22/CD30/CD38/ CD70/CD123	I/II	Unknown	100	NA	NA	NA	China	NCT03125577
Identify new potential targets for CAR-T cell therapy	BCMA,CD19,CD22, CD33, CD38, c-Met, DR5, EGFR, MSLN, NY-ESO-1	I/II	Unknown	73	NA	NA	NA	China	NCT03638206
	CD19	NA	Terminated	4	NA	NA	NA	US	NCT03436771
	CD19 (SENL-B19)	I/II	Unknown	50	NA	NA	NA	China	NCT03312205
	CD19 and/or BCMA	I	Unknown	10	NA	NA	NA	China	NCT03767725
	CD19	II	Terminated	6	NA	NA	NA	US	NCT02794246
	SLAMF-7	I	Recruiting	30	NA	NA	NA	China	NCT03710421
	SLAMF-7	I	Completed	24	NA	NA	NA	US	NCT03958656
	SLAMF-7 (UCARTCS1A)	I (MELANI-01)	Terminated	11	NA	NA	NA	US	NCT04142619
	SLAMF-7	I/II (CARAMBA-1)	Recruiting	38	NA	NA	NA	Germany	NCT04499339
	CD38	I	Terminated	10	NA	NA	NA	US	NCT03464916
	CD138	I	Recruiting	33	NA	NA	NA	US	NCT03672318
	CD138 (CART-138)	I/II	Recruiting	10	0/0	80	NA	China	NCT01886976
	GPRC5D (OriCAR-017)	Early I	Not Recruiting	15	0/0	100	11.37	China	NCT05016778 [87]
	GPRC5D	I/II	Recruiting	18	0/0	85.7	Not yet reached	China	NCT05739188 [88]
	GPRC5D	I/II	Recruiting	18	NA	NA	NA	China	NCT05749133
	GPRC5D	I	Recruiting	40	NA	NA	NA	China	NCT06084962
	GPRC5D	I	Recruiting	12	NA	NA	NA	China	NCT05759793
	GPRC5D (BMS-986393, CC-95266)	II	Recruiting	180	6/0	89	Not yet reached	US	NCT04674813 [89]
	GPRC5D	I	Recruiting	18	NA	NA	NA	China	NCT05219721
	GPRC5D (MCARH109)	I	Recruiting	17	6/6	71	Not yet reached	US	NCT04555551 [90]
	CD44v6	I/II	Terminated	8	NA	NA	NA	Italy	NCT04097301
	NKG2D (CYAD-01)	I (CM-CS1)	Completed	12	0/0	NA	NA	US	NCT02203825 [91]
	NKG2D (CYAD-01)	I/II (THINK)	Unknown	146	31/NA	NA	NA	US	NCT03018405 [31]
	TnMUC1	I	Terminated	16	NA	NA	NA	US	NCT04025216
	CD70	Early I	Recruiting	108	NA	NA	NA	China	NCT04662294
	MMG49	I/II	Recruiting	49	NA	NA	NA	Japan	NCT04649073
	Kappa-light chain (κ.CARTs)	I (CHARKALL)	Recruiting	54	NA	NA	NA	US	NCT00881920
Immune checkpoint inhibitor (Pembrolizumab)	BCMA	II	Terminated	25	NA	NA	NA	US	NCT05191472
Immune checkpoint inhibitor (Pembrolizumab)	BCMA	II	Recruiting	30	NA	NA	NA	US	NCT05204160
Immune checkpoint inhibitor (Nivolumab)	BCMA	II	Not recruiting	20	NA	NA	NA	US	NCT04205409
Immune checkpoint inhibitor(secreting PD-1 Fc)	BCMA (BCMA-PD1)	II	Recruiting	30	NA	NA	NA	China	NCT04162119
Co-express CXCR4	BCMA	Early I	Recruiting	12	NA	NA	NA	China	NCT04727008

B-cell maturation antigen (BCMA), Cluster of differentiation (CD), Signaling lymphocyte activation molecule family-7 (SLAMF-7), Natural-killer group 2 member D (NKG2D), G protein-coupled receptor class C group 5 member D (GPRC5D), A proliferation-inducing ligand (APRIL), Transmembrane activator and calcium-modulating cyclophilin ligand interactor (TACI), Tn antigen on membrane mucin 1 (TnMUC1), Epidermal growth factor receptor (EGFR), Cellular-mesenchymal-epithelial transition factor (c-Met), Mesothelin (MSLN), New York esophageal squamous cell carcinoma-1 (NY-ESO-1), C-X-C chemokine receptor type 4 (CXCR4), Cytokine release syndrome (CRS), Immune effector cell-associated neurotoxicity syndrome (ICANS), Overall response rate (ORR), and Progression-free survival (PFS)

Moreover, the exploration of new targets, such as Fc receptor-homolog 5 (FcRH5) (also known as FcRL5, IRTA2, or CD307), can potentially expand the repertoire of CAR-T therapies for MM [92]. The following sections provide an overview of various myeloma antigens extensively investigated, either as single therapies or in combination as dual or multi-target strategies. These approaches are designed to address resistance issues observed in BCMA-targeted CAR T-cell therapies.

##### Targeting SLAMF-7

SLAMF-7, also known as CS1, is found in cancer-associated fibroblasts (CAFs), the predominant immunosuppressive components in the tumor microenvironment that promote MM cell proliferation [93]. Autologous SLAMF-7 targeting CAR-Ts (NCT04499339, NCT03958656, NCT03710421), allogeneic SLAMF-7 targeting CAR-Ts (NCT04142619), and BCMA/SLAMF-7 targeting CAR-Ts (NCT04662099, NCT04156269) have been engineered for R/R MM patients [54, 71, 84, 93].

##### Targeting GPRC5D

GPRC5D, a largely myeloma-specific orphan receptor with limited expression in other tissues, has been proposed as a valuable immunotherapeutic target for managing MM. Clinical trials are underway (NCT04674813, NCT05016778, NCT05739188, NCT05749133, NCT06084962, NCT05759793, NCT05219721) to assess autologous CAR T-cells targeting GPRC5D in R/R MM patients [88, 89, 94]. Additionally, there are CAR-T therapies targeting both BCMA and GPRC5D (NCT05431608, NCT06068400, NCT05325801, NCT05998928, and NCT05509530) [90, 95]. Moreover, MCARH109 (NCT04555551) has been administered to heavily treated MM patients, including those who relapsed after BCMA CAR T-cell therapy [90].

It is noteworthy that recent research has linked relapses in MM patients receiving anti-GPRC5D CAR T to focal biallelic deletions at the GPRC5D locus, while studies have observed the selective expansion of pre-existing subclones with biallelic GPRC5D loss, highlighting potential mechanisms of resistance to this targeted therapy [96, 97].

##### Targeting integrin β7

MM expresses high levels of integrin β7 receptor subfamily, contributing to adhesion, migration, homing, invasion, and drug resistance. CARs targeting the integrin β7 receptor are currently being evaluated in a multitarget setting (NCT03778346) [98].

Recently, a new generation of CARs has been developed targeting MMG49, an inducible epitope located within the N-terminal region of the integrin β7 chain in MM. Safety and effectiveness assessments for OPC-415 (a CART cell targeting MMG49) are also underway in another study (NCT04649073) [65].

##### Targeting NKG2D

Stress-induced ligands, including MHC-I chain-related proteins (MIC) like MICA and MICB, and structurally diverse unique long 16-binding proteins 1 to 6 (UL16-binding proteins or ULBPs), are typically upregulated in various hematological malignancies. When these ligands bind to NKG2D receptors on cytotoxic immune cells, such as NK or T cells, they form a hexameric structure with the adaptor protein DAP10 (DNAX-activating protein of 10 kDa). This interaction is crucial for eliminating tumor cells [65].

Consequently, CYAD-01, an autologous NKG2D-CAR T cell, was developed using a full-length natural NKG2D receptor, termed DAP10-NKG2D CAR, which is fused to the CD3ζ intracellular domain without any additional stimulation domains (NKG2D-CD3ζ CAR) [67, 91]. Encouraging outcomes observed in completed primary investigations (CM-CS1) involving CYAD-01 (NCT02203825) paved the way for a phase I clinical trial (THINK) (NCT03018405) [31].

##### Targeting TnMUC1

The membrane mucin 1 (MUC1) glycoprotein, primarily found on the luminal surface of the simple and glandular epithelium as well as on leukocytes, becomes altered in tumors. The TnMUC1 protein, marked by abnormal glycosylation, is often overexpressed in these tumors. CAR-TnMUC1, designed to target the Tn glycan epitope of MUC1, incorporates a transmembrane CD8α region along with two intracellular signaling domains, CD2 and CD3ζ. In a phase I study (NCT04025216), it was observed that CD2 signaling led to reduced exhaustion and prolonged persistence of T cells within the tumor microenvironment [99]. However, this trial has since been terminated.

##### Targeting BCMA/TACI

**Fig. 3:**
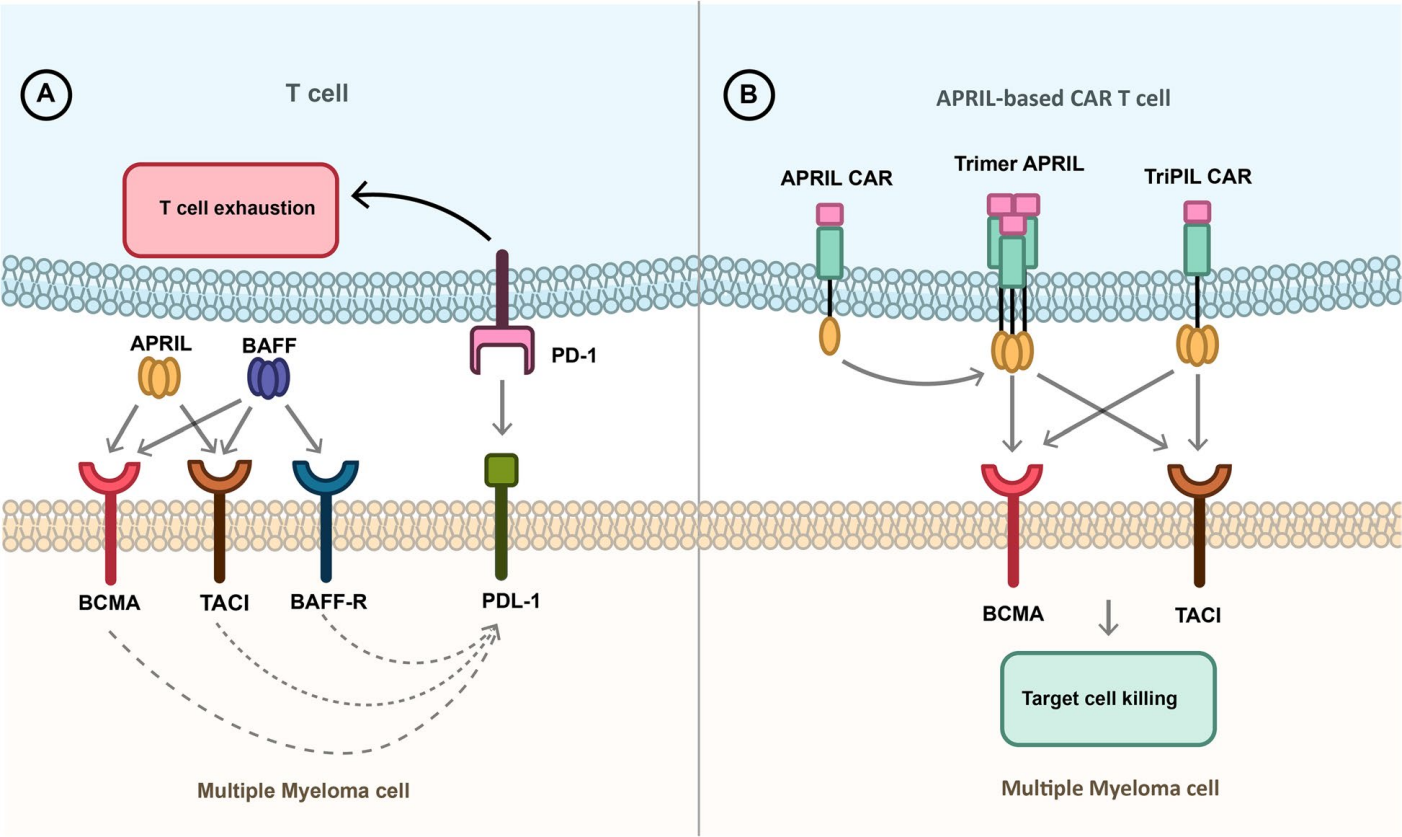
APRIL-based CAR T-cells. **A** APRIL and BAFF bind to BAFF-R, TACI, and BCMA receptors which leads to the expression of PDL-1 on the cancer cell and induces T cell exhaustion usingof PD-1 on the T-cell surface. **B** APRIL-base CARs can bind to the BCMA and TACI expressing multiple myeloma cells and inducing tumor shrinkage. The APRIL CAR should create a trimer protein to bind to the receptor, similar to APRIL binding to its target as a trimer protein. However, in TRiPIL CAR, an APRIL trimer protein serves as the binding domain so that oligomerization is not required to bind to receptors. TriPIL CAR is a superior choice for targeting MM cells

B-cell activating factor (BAFF) and a proliferation-inducing ligand (APRIL) are recognized as pivotal signaling molecules contributing to MM resistance [100]. They bind to BCMA, BAFF-R, and transmembrane activator and calcium-modulating cyclophilin ligand interactor (TACI) receptors, triggering expression of programmed cell death-ligand 1 (PD-L1) and exhausting T cells [101, 102]. The biological foundation of these molecules has spurred the creation of APRIL-CAR, a CAR that relies on a natural ligand instead of employing a scFv derived from an antibody. TACI and BCMA are targeted simultaneously in BCMA/TACI-based CARs to address heterogeneous antigens [101].

consequently, april car t-cells, including auto2 (nct03287804), APRIL (NCT04657861), and TriPRIL (NCT05020444), were developed to improve their binding capacity [103]. While AUTO2 was initially promising in preclinical and clinical trials, it was recently terminated due to concerns about durability and relapses in many patients [85]. On the other hand, TriPRIL, with its trimeric structure, has demonstrated superior binding capacity in initial studies compared to monomeric APRIL-based CAR-Ts [85, 103] (Fig. 3).

#### Multi-antigen targeting to address heterogeneity and overcome BCMA evasion

**Fig. 4:**
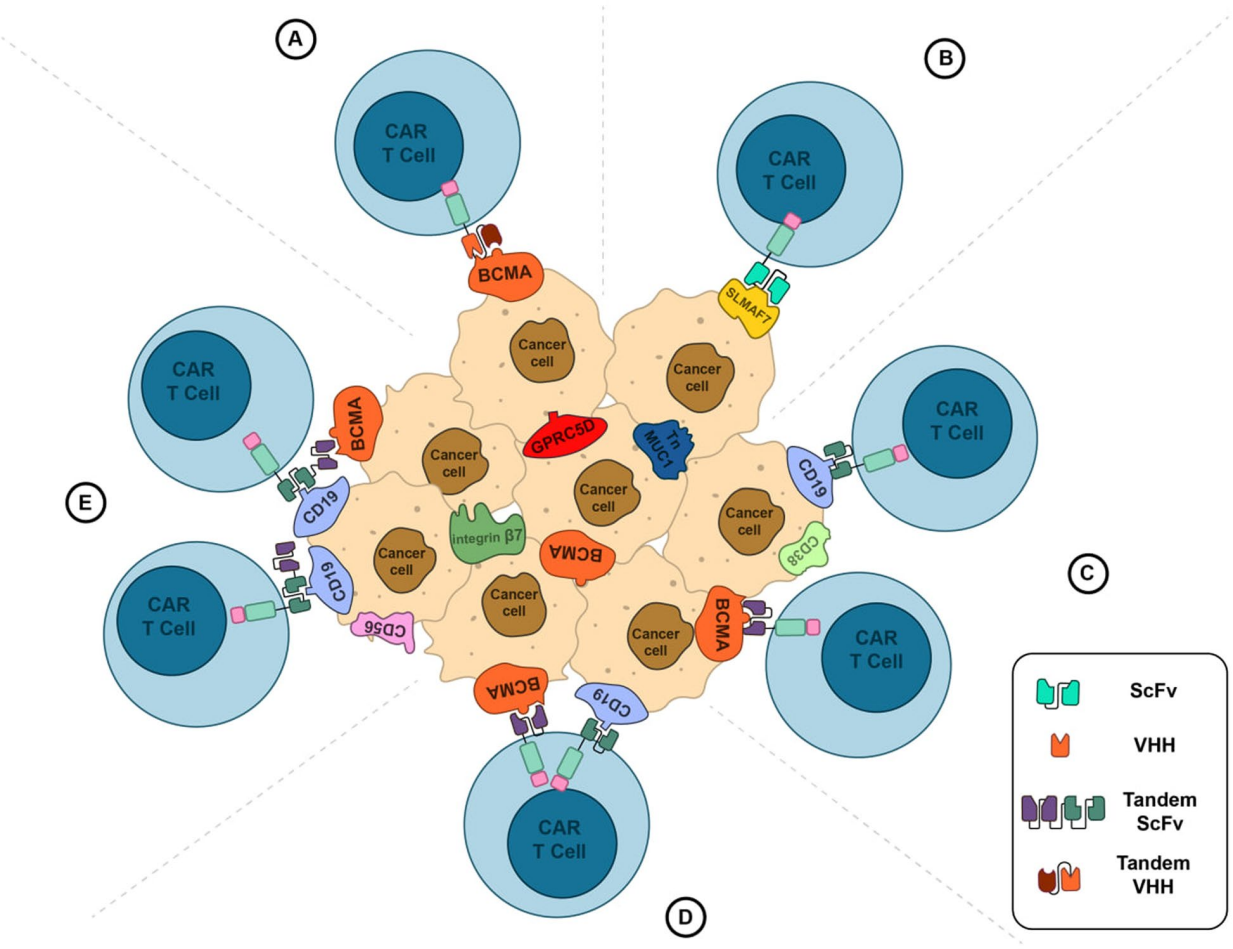
Managing tumor heterogeneity. **A** VHH-based CAR targeting two epitopes of the same antigen. **B** CAR T-cell targeting novel antigens like SLAMF7 **C**. Co-infusion of two distinct CAR T-cells targeting different antigens. **D** CAR T-cell expression two distinct CARs targeting different antigens. **E** Bispecific CAR T-cells targeting two different antigens. Recognition of each target antigen leads to T-cell activation and tumor shrinkage

Prominent recent trials aiming to target dual or multiple antigens simultaneously on tumor cell surfaces typically utilize products combining a range of antigens. These combinations include BCMA, CD19, and CD138 (NCT03196414); BCMA, CD38, CD138, and CD56 (NCT03473496, NCT03271632); BCMA, CD38, CD138, SLAMF-7, and Integrin β7 (NCT03778346); and CD19, CD20, CD22, CD30, CD38, CD70, and CD123 (NCT03125577). These trials employ CAR-T therapies with bispecific targeting domains, tandem expression of both domains, or the co-infusion of distinct CAR-Ts targeting different antigens (Fig. 4) (Table 2). These innovative approaches are designed to address the heterogeneity of multiple myeloma and combat BCMA evasion or shedding by targeting a diverse array of surface antigens. [65, 104].

After identifying a suitable target antigen, the next major challenge lies in enabling CAR T-cells to efficiently penetrate tumors characterized by dense barriers and a hostile microenvironment.

#### Utilizing gamma-secretase inhibitor to augment BCMA-Targeted CAR T-cell therapy

The high expression of Notch pathway receptors and ligands in MM cells and the bone marrow environment may explain their role in MM progression [105]. Notch signaling promotes cell proliferation and drug resistance through cell fate determination, a critical aspect of cell–cell communication. It acts through both homotypic activation, where MM cells interact via their own receptors and ligands, and heterotypic activation, involving interactions with bone marrow stromal cells, osteoclasts, and osteocytes [106].

Notch signaling regulates the CXCR4/SDF1α chemokine system and facilitates MM cell migration to the bone marrow [106]. This pathway also promotes MM cell proliferation by upregulating cyclin D1, hairy and enhancer of split-1 (HES1), and hairy/enhancer-of-split related with YRPW motif protein (HEY1) [105]. Notch receptor interacts with other pathways such as PI3K/AKT and NF-κB, further boosting MM cell survival and proliferation [105, 106].

Nirogacestat, a small-molecule gamma-secretase inhibitor (GSI), primarily inhibits Notch signaling and enhances the efficacy of BCMA-targeted therapies [107]. By blocking the proteolysis of Notch receptors, GSIs can potentially slow tumor growth and increase the susceptibility of MM cells to other treatments [108]. GSIs exhibit synergistic effects, enhancing the cytotoxic effects of other MM treatments, such as bortezomib [109]. Combining GSIs with traditional chemoradiotherapies can potentially overcome resistance mechanisms.

GSIs may induce differentiation and apoptosis in cancer stem-like cells, which are often responsible for treatment resistance and relapse in MM [110]. Additionally, by modulating the tumor microenvironment, GSIs can alter the interactions between MM cells and the bone marrow microenvironment, which is crucial for MM progression. [111, 112].

Moreover, GSIs increase the presence of BCMA on the cell surface and reduce the levels of soluble BCMA (sBCMA) by preventing the cleavage and shedding of BCMA from the surface of myeloma cells. GSIs can maintain the increased BCMA surface density for an extended period, potentially prolonging the effectiveness of CAR-T cell therapy with minimal impact on T cells [113].

Nirogacestat’s effectiveness has been assessed in several clinical trials involving “off-the-shelf” CAR T-cell products, as seen in studies such as NCT04093596 and NCT04171843 [38]. Another clinical trial utilized Crenigacestat (LY3039478; JSMD194) as a gamma-secretase inhibitor for MM patients with ide-cel in Phase I/II (KarMMa-7) (NCT04855136) and with anti-BCMA CAR T-cells (NCT03502577) [110]. (Table 4).

### Immunosuppressive tumor microenvironment

Although multiple myeloma is often classified as blood cancer, uncontrolled plasma cell proliferation can lead to tumor formation, either within the bones (solitary plasmacytoma) or outside the bones (extramedullary plasmacytoma) [114]. A significant characteristic of MM is the creation of a favorable tumor microenvironment (TME), where local immunosuppression plays a critical role in continuously stimulating antigens [17].

The TME in MM is composed of a diverse array of cellular and acellular elements that contribute to the functional decline, exhaustion, senescence, and removal of CAR T-cells [115]. These include cellular components like regulatory T cells, bone marrow stromal cells (BMSCs), myeloid-derived suppressor cells (MDSCs), tumor-associated macrophages (TAMs), and tumor-associated neutrophils (TANs), in conjunction with various acellular elements like cytokines such as IL-6, transforming growth factor-β (TGF-β), IL-10, tumor-derived granulocyte–macrophage colony-stimulating factor (GM-CSF) and vascular endothelial growth factor (VEGF), proteins like programmed death-ligand 1 (PD-L1), indoleamine 2,3-dioxygenase (IDO), and CD38, and chemicals like prostaglandin E2 (PGE2) and Adenosine [16, 115]. Additionally, environmental conditions such as low extracellular pH (acidosis), limited nutritional resources, and low oxygen levels (hypoxia) further exacerbate the challenges faced by CAR T-cells within this microenvironment [116].

Enhancing our understanding of tumor-induced CAR T-cell suppression is critical for advancing immunotherapy, and various strategies are available to bolster CAR T-cells in this challenging microenvironment.

#### Surmounting immune checkpoints in CAR T-cell therapy

GM-CSF in the TME leads to MDSCs expansion, triggering signal transducer and activator of transcription 3.(STAT3)-mediated programmed death-ligand 1 (PD-L1) induction in them. PD-L1 directly interacts with PD-1 on CAR T-cells, leading to their suppression [117]. Moreover, the hypoxic conditions in the TME induce T cell anergy and exhaustion by increasing the expression of immune checkpoint molecules such as PD-1, cytotoxic T-lymphocyte associated protein 4 (CTLA4), lymphocyte activation gene 3 (LAG3), T-cell immunoglobulin mucin-3 (TIM-3), B and T lymphocyte attenuator (BTLA), and T cell immunoreceptor with Ig and ITIM domains (TIGIT), hindering their activation and cytotoxicity [118]. Furthermore, a significant increase in immunosuppressive regulatory T cells plays a pivotal role in maintaining immune tolerance by releasing immunosuppressive cytokines like TGF-β and IL-10 and expressing immune-inhibitory molecules such as PD1, TIM3, and CD38 [16, 118].

Approved mAbs targeting immune checkpoints can enhance CAR T-cell therapy. These include Ipilimumab for CTLA-4; Dostarlimab, Pembrolizumab, Cemiplimab, and Nivolumab for PD-1; and Atezolizumab, Durvalumab, and Avelumab for PD-L1 [119–123]. While current research provides valuable insights into the mechanisms and potential of these monoclonal antibodies targeting immune checkpoints, a select few have progressed to late-phase studies or limited clinical use in specific MM patient populations. Integrating these agents with CAR T-cell therapy represents a promising frontier in MM treatment, though their optimal application remains an active area of research. (Table 2).

However, researchers have discovered that directly disrupting the genes encoding these checkpoints through genomic editing technologies yields comparable improvements in CAR T-cell growth and cytotoxicity within the TME. CAR T-cells with genetic modifications, disrupting PD-1, TGF-β, CTLA-4, LAG3, or TIGIT genes, demonstrate an impressive ability to suppress target cells while retaining their essential T-cell characteristics [123–126]. Likewise, by knocking out the GM-CSF gene through techniques like TALEN or CRISPR/Cas9, CAR T-cells not only experience an enhancement in their cytotoxic activity but also a suppression in the secretion of CRS related biomarkers like IL-6 and IL-8 [127, 128].

Armored CAR T-cells are also engineered to combat the immunosuppressive TME through various innovative strategies. Some armored CAR T-cells are designed to express cytokines or ligands like IL-7, IL-12, IL-15, IL-18, and CD40L, which boost T-cell proliferation, survival, and persistence within the tumor [129]. Others express anti-apoptotic factors from the BCL-2 family of proteins, like BCLxL (BCMA-BCL2L1), to enhance T-cell survival [130]. Additionally, some armored CAR T-cells are engineered to express enzymes such as heparanase, which degrades the extracellular matrix to improve tumor infiltration, thereby protecting against oxidative stress in the TME [131]. Specific therapeutic approaches such as pro-survival gene modifications, including AKT overexpression, have the potential to improve CAR T-cell function in nutrient-poor conditions [132, 133]. Metabolic enhancements, such as overexpressing phosphoenolpyruvate carboxykinase 1 (PCK1) or glutamine synthetase, can allow CAR T-cells to thrive in glucose- or glutamine-depleted environments [134].

Additionally, some armored CAR T-cells are engineered to secrete antibodies targeting PD-1 or PD-L1, effectively preventing the activation of this immunosuppressive pathway [135–137] An investigation is underway using anti-BCMA CAR T-cells to produce mutant PD-1 Fc fusion protein in R/R MM patients (NCT04162119) (Table 2).

#### Improving CAR T-cell trafficking

Recent research has explored utilizing the CXCL12-CXCR4 chemokine axis to improve the intratumoral trafficking of adoptive T cells [138]. In an effort to enhance the homing of CAR T-cells into the CXCL12-rich bone marrow, BCMA-targeted CAR T-cells were engineered to co-express CXCR4. A clinical trial for multiple myeloma (NCT04727008) demonstrated that this approach led to a reduction in tumor burden [139] (Table 2). Additionally, preclinical studies showed that concurrent CXCR4 and anti-BCMA CAR expression on NK cells effectively inhibited MM progression in mouse models [140].Ulocuplumab, the first mAb targeting CXCR4, has shown promise in a phase Ib/II study focused on R/RMM [141].

However, enhancing CAR T-cells’ effectiveness may lead to increased inflammatory signals and associated toxicities. Therefore, precise immunomodulation is crucial for the future application of CAR T-cells in multiple myeloma treatment, aiming to enhance control over the tumor microenvironment while minimizing potential adverse effects.

## Clinical considerations and safety management in CAR T-cell therapy for multiple myeloma

### Addressing cytotoxicity challenges in CAR T-cell therapy

While CAR-T therapies have demonstrated remarkable therapeutic outcomes in R/R MM, they can also lead to significant toxicities. [142, 143]. The two main complications are cytokine release syndrome (CRS) and immune effector cell-associated neurotoxicity syndrome (ICANS), caused by the release of inflammatory cytokines such as TNF-α, IFN-γ, GM-CSF, IL-1, IL-2, IL-6, IL-8, and IL-10, as well as immune responses mediated by other innate immune cells, including immune effector cell-associated hemophagocytic lymphohistiocytosis-like syndrome (IEC-HS) and macrophage activation cells (MAC). These processes often form a self-aggravating cycle [144–146].

CRS typically occurs within 1 to 7 days post-infusion of CAR T-cells, presenting with symptoms ranging from mild flu-like symptoms to severe organ failure, hypotension, hypoxia, and even cardiac arrest in extreme cases.Tumor lysis syndrome (TLS) can also develop in hematological malignancies, presenting symptoms that overlap with those of CRS [147]. The severity of CRS is typically graded on a scale from 1 to 5. Most CRS events are classified as mild(grade 1 or 2), while severe cases are designated as grade 3 or 4. Grade 5, representing the highest severity level, is rarely observed [148].

‘ICANS can occur concurrently with or independently of CRS and typically occurs 3 to 8 days after CAR T-cell infusion. Neurotoxicity can range from mild headaches or delirium to severe confusion, apraxia, or seizures [149].

For cilta-cel, CRS occurred in approximately 95% of patients, but only 4.1% of these cases were severe. ICANS was observed in about 16.5% of patients at any grade, with severe cases reported in 2.1% of patients [150]. In the case of ide-cel, the incidence of CRS was also about 84% of patients, with severe CRS occurring in approximately 5%. ICANS was observed in about 17% of patients at any grade, with severe cases in 3% of patients [151]. Although currently, studies are underway to reduce the mentioned side effects [152].

Although at lower levels, desired antigens like BCMA are also expressed by some normal cells. Consequently, CAR T-cell therapy targeting these antigens may lead to the elimination of healthy cells, a phenomenon known as “on-target off-tumor” toxicities.. This can result in complications such as B-cell aplasia, cytopenia, hypogammaglobulinemia, and immunosuppression, which may increase patients’ susceptibility to various types of infectious diseases [153].

Researchers have identified a correlation between the number of prior therapies, the occurrence of CRS, and the extent of bone marrow tumor burden. These factors were associated with prolonged hematological recovery periods. Patients experiencing severe CRS had lower neutrophil counts, hematocrit levels, and platelet nadirs, necessitating more platelet and red cell transfusions compared to those with mild CRS [20].

Additionally, in MM patients, research has shown that soluble BCMA (sBCMA) levels correlate with laboratory evaluations of tumor burden and prognosis. Elevated sBCMA levels are associated with worse outcomes and a higher incidence of adverse events, including CRS and ICANS) [154]. Currently, the measurement of sBCMA is not standard practice in clinical settings. Instead, most clinicians rely on other assessments of tumor burden, including bone marrow plasma cell percentage, β2 microglobulin levels, serum protein electrophoresis (SPEP)/M-spike, and values associated with light chain disease. However, sBCMA shows potential for patient stratification in clinical trials and real-time disease progression monitoring [155].

To enhance safety, recent studies have explored various CAR T-cell strategies, such as reducing the number of CAR T-cells administered to patients, combining small molecule drugs with CAR T-cells, or mitigating toxicity by engineering CAR T-cells with suicide genes (Table 3).

**Table 3:** Clinical trials on safety management in CAR-T therapy for multiple myeloma

						Adverse effect	Outcomes			
Target / CAR-T Name	Mechanism of Action	Combination. Regimen	Phase/ Study Name	Status	No. of Patient	Grade ≥ 3 CRS/ICANS,%	ORR,%	Median PFS, months	Location	NCT
BCMA (Ide-cel)	IL-6RA	Tocilizumab	I	Completed	67	6.5/1.6	75.8	8.8	US	NCT02658929 [156]
BCMA (Ide-cel)	IL-6RA	Tocilizumab	II (KarMMa)	Completed	149	5/3	73	8.6	US	NCT03361748 [77]
BCMA (Cilta-cel)	IL-6RA	Tocilizumab	I/II CARTITUDE-1	Completed	126	4/10	98	34.9	US	NCT03548207 [11]
BCMA (Cilta-cel)	IL-6RA	Tocilizumab	I/II (LEGEND-2)	Not recruiting	100	9.5/0	87.8	18	US	NCT03090659 [157]
BCMA	IL-6RA	Tocilizumab	I	Completed	30	38/19	81	7.8	US	NCT02215967 [158]
BCMA (Bb21217)	IL-6RA	Tocilizumab	I (CRB-402)	Completed	72	4/7	55	11.9	US	NCT03274219 [30]
BCMA/TACI (AUTO2)	IL-6RA	Tocilizumab	I/II	Terminated	12	0/0	45.5	NA	UK	NCT03287804 [85]
BCMA (UPenn)	IL-6RA Human	Tocilizumab scFv	I	Completed	25	32/12	48	2.7	US	NCT02546167 [72]
BCMA	IL-6A	Siltuximab	II	Recruiting	30	NA	NA	NA	US	NCT04975555
CD19/BCMA	LCK inhibitor	Dasatinib	Early I	Recruiting	120	NA	NA	NA	China	NCT04603872
BCMA (Orva-cel; JCARH125)	IL-1RA Human	Anakinra scFv	I/II (EVOLVE)	Completed	165	2/4	91	NA	US	NCT03430011 [29]
CD44v6	HSV-TK (suicide gene)	GCV	I/II	Terminated	58	NA	NA	NA	Italy	NCT04097301
BCMA (P-BCMA-101)	iCasp9 (suicide gene) Humanized	Rimiducid scFv	I/II	Terminated	105	2/0	57	NA	US	NCT03288493 [36]
BCMA (P-BCMA-101)	iCasp9 (suicide gene) Humanized	Rimiducid scFv	I	Not recruiting	100	NA	NA	NA	US	NCT03741127
BCMA (P-BCMA-ALLO1)	iCasp9 (suicide gene) Humanized	Rimiducid scFv	I (MM)	Recruiting	135	0/0	82	Not yet reached	US	NCT04960579 [37]
CD19 and CD20/CD22/ CD30/CD38/ CD70/CD123	iCasp9 (suicide gene)	Rimiducid	I/II	Unknown	100	NA	NA	NA	China	NCT03125577
SLAMF-7	iCasp9 (suicide gene)	Rimiducid	I	Completed	24	NA	NA	NA	US	NCT03958656
BCMA/CD19	EGFRt/ suicide gene	Cetuximab	I/II	Recruiting	15	0/0	100	NA	China	NCT03455972 [82]
BCMA (MCARH171)	EGFRt (suicide gene) Human	Cetuximab s scFv	I	Not recruiting	20	2/0	64	Not yet reached	US	NCT03070327 [159]
BCMA (HDS269B)	EGFRt (suicide gene) Human	Cetuximab scFv	I	Recruiting	10	6.12/0	77	10	China	NCT03093168 [160]
BCMA	EGFRt (suicide gene) Human	Cetuximab scFv	Early I	Terminated	18	NA	NA	NA	US	NCT03502577 [110]
BCMA	Humanized	scFv	Early I	Not yet recruiting	50	NA	NA	NA	China	NCT04670055
BCMA (ARI0002h)	Humanized	scFv	I/II	Recruiting	73	5/0	95	20	China	NCT04309981 [161]
BCMA/CD19	Humanized	scFv	I	Unknown	20	NA	NA	NA	China	NCT04194931
BCMA (CT103A)	Human	scFv	I	Not yet recruiting	12	NA	NA	Not yet reached	US	NCT05698303
BCMA (CT103A)	Human	ScFv	I	Unknown	20	NA	NA	NA	China	NCT05201118
BCMA (CT103A)	Human	scFv	I (FUMANBA-1)	Recruiting	132	1/0	96	Not yet reached	China	NCT05066646 [162]
BCMA (CT103A)	Human	scFv	I	Not yet recruiting	20	NA	NA	Not yet reached	China	NCT05181501
BCMA (FCARH143)	Human	scFv	I	Completed	21	0/0	100	NA	US	NCT03338972 [28]
BCMA (CT053; Zevor-cel)	Human	scFv	I/II (LUMMICAR STUDY 1)	Recruiting	114	6/3	100	25	China	NCT03975907 [163]
BCMA (CT053; Zevor-cel)	Human	scFv	I/II (LUMMICAR STUDY 2)	Not recruiting	105	0/0	100	Not yet reached	US	NCT03915184 [164]
BCMA (C-CAR088)	Human	scFv	I	Unknown	12	3/0	96.4	Not yet reached	China	NCT03815383 [165]
BCMA (CAR-NK)	Human	scFv	I	Recruiting	18	NA	NA	NA	China	NCT06045091
BCMA	Human	scFv	I	Recruiting	18	2/0	71.4	8.9	China	NCT04003168 [166]
BCMA (ddBCMA CAR-T)	Peptide	scFv	I	Recruiting	65	1/1	90	Not yet reached	US	NCT04155749 [167]
BCMA (FHVH33)	Human	VH	I	Not recruiting	35	1/0	NA	NA	US	NCT03602612 [168]
BCMA	Nanobody	VHH	I	Unknown	15	2.9/0	88.2	12.1	China	NCT03661554 [169]
BCMA	Nanobody	VHH	I	Unknown	15	NA	NA	NA	China	NCT03664661

IL-1 receptor antagonist (IL-1RA), IL-6 receptor antagonist (IL-6RA), Lymphocyte-specific protein tyrosine kinase (LCK), Herpes simplex virus thymidine kinase (HSV-TK), Inducible caspase 9 (iCasp9), Truncated epidermal growth factor receptor (tEGFR/EGFRt), Single-chain variable fragments (scFv), variable heavy chain(VHH), human heavy-chain-only binding domain (VH), d-domain-based antigen binder B-Cell Maturation Antigen (ddBCMA), Cytokine release syndrome (CRS), Immune effector cell-associated neurotoxicity syndrome (ICANS), Overall response rate (ORR), and Progression-free survival (PFS)

#### Combining pharmacological immunosuppressive agents

Numerous studies have investigated drugs aimed at neutralizing the toxicity associated with CAR T-cell therapy in multiple myeloma. Tocilizumab, a mAb against IL-6 receptors (IL-6RA), and corticosteroids are traditionally used to manage toxicities (NCT02215967, NCT02546167, NCT02658929, NCT03090659, NCT03274219, NCT03548207, NCT03361748, NCT03287804) [142]. For ICANS and more severe forms of CRS, corticosteroids are beneficial since Tocilizumab does not penetrate the blood–brain barrier effectively [170] (Table 3).

Clinical trials have revealed that while Tocilizumab effectively mitigated cytotoxicity without compromising CAR T-cell efficacy, approximately 30% of patients still experienced severe grade 3 or higher CRS/ICANS. Even the early administration of steroids failed to alleviate around 13% of the severe cases [171, 172]. Therefore, Tocilizumab-refractory CRS or steroid-refractory ICANS patients require novel treatments [173].

Anakinra, an FDA-approved recombinant IL-1 receptor antagonist (IL-1RA) protein typically used to treat auto-inflammatory diseases, has shown promise in managing CAR T-cell therapy-associated toxicities [172, 174, 175]. For instance, a Phase II study assessing the efficacy of Orva-cel (NCT03430011) included a group of patients given prophylactic Anakinra treatment, which substantially reduced CRS levels following infusion [29]. Recent consensus guidelines suggest Anakinra as a third-line agent for refractory CAR T-associated toxicities [172].

Dasatinib, an inhibitor of lymphocyte-specific protein tyrosine kinase (LCK), blocks the phosphorylation of CD3ζ and ZAP70 [176]. It protects CAR-Ts from lethal CRS by limiting cytolytic activity, cytokine production, and expansion, potentially serving as a pharmacologic on/off switch for CAR T-cells [18]. Dasatinib has been shown to ameliorate the exhausted phenotype of CAR T-cells by upregulating memory-associated genes (such as TCF7 and CCR7) and downregulating the immune checkpoint molecule like PD1, as well as exhaustion-related regulators (such as NR4A1, BATF3, ATF4, and FO) [177]. A Phase I clinical trial (NCT04603872) is recruiting R/R MM patients for CAR T-cells targeted at CD19/BCMA in combination with Dasatinib [80]. In addition, Siltuximab, an anti-IL-6 mAb, has been used in multiple myeloma CAR T-cell trials for severe cases of CRS/ICANS (NCT04975555) (Table 3).

The ZUMA-19 trial (NCT04314843) is investigating the use of Lenzilumab, an anti-human GM-CSF neutralizing mAb, for Large B-cell lymphoma to assess its efficacy and safety in preventing or minimizing CRS/ICANS in combination with CAR T-cell therapy. This aligns with the FDA guidance on prospective registration phase 3 studies for various CAR T-cell assessments [178].

#### Directly addressing CAR T-cell toxicity

**Fig. 5:**
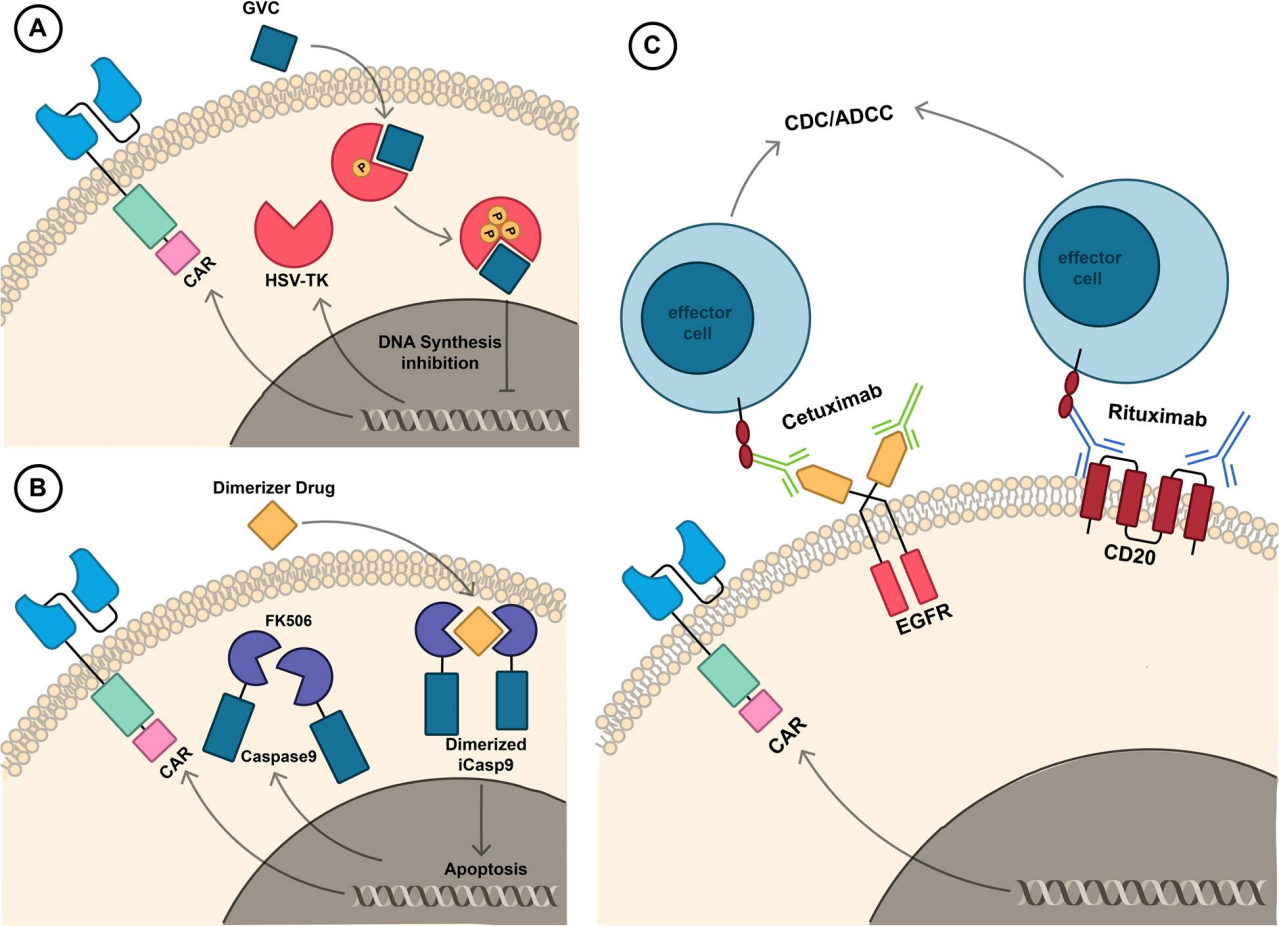
Approaches to mitigating the toxicity linked with CAR T-cell therapy directly. **A** The HSV-TK safety switch and its mode of action. Ganciclovir (GCV) administration leads to HSV-TK gene activation and its conversion to GCV-monophosphate (MP) and eventually further phosphorylated to convert to a toxic GCV-trisphosphate (TP). GCV-TP disrupts DNA synthesis, selectively eliminating CAR T cells. **B** The inducible caspase 9 (iCasp9) suicide switch and its mechanism. Dimerizing drug administration induces iCasp molecules (FK506) to form dimers, activating downstream apoptotic cascades, and resulting in CAR T cell death. **C** Examples of mAb-based safety switches and their functions. Co-expression of CAR and cell surface proteins such as EGFRt and CD20 facilitates selective CAR T cell elimination through pharmaceutical-grade mAbs like Cetuximab and Rituximab, which engage immune effector cells or complement fixation cells via mechanisms such as antibody-dependent cell-mediated cytotoxicity (ADCC) and complement-dependent cytotoxicity (CDC) upon specific mAb introduction

Suicide genes are genetic elements that allow for the permanent removal of introduced cells through the use of pharmaceutical agents. This removal is achieved via metabolic processes that transform non-toxic substances into toxic agents, primarily when unexpected adverse reactions arise. Prominent instances of inducible suicide genes include herpes simplex virus thymidine kinase (HSV-TK) and recombinant human inducible caspase 9 (iCasp9/iC9). Additionally, the expression of truncated epidermal growth factor receptor (tEGFR/EGFRt) or CD20 molecular proteins are utilized to selectively eliminate CAR T-cells that express these genes when necessary [179, 180] (Fig. 5).

##### HSV-TK

In cases of severe toxicity, a non-toxic prodrug, ganciclovir (GCV), can be administered to selectively activate the HSV-TK gene within transplanted CAR T-cells. This is due to HSV-TK’s high affinity for GCV, which mammalian cell thymidine kinase lacks [181]. Upon HSV-TK phosphorylation of GCV, GCV-monophosphate (MP) eventually converts to a toxic GCV-trisphosphate (TP). This toxic form competitively inhibits guanosine trisphosphate (dGTP), resulting in the cessation of DNA chain synthesis and the demise of specific proliferating cells [182, 183]. The HSV-TK-GCV suicide switch system also activates caspases through ligand-independent aggregation of CD95 and the formation of Fas-associated death domain protein (FADD) and TNF-R-mediated apoptosis in CAR T-cells [184].

Numerous clinical trials have explored CAR-Ts containing the HSV-TK gene for hematological malignancies.A Phase I/II clinical trial investigated the HSV-TK Mut2 gene in anti-CD44v6 CAR T-cells in R/R MM patients (NCT04097301) [185]. However, this trial has since been terminated [181] (Table 3).

##### iCasp9

The other suicide gene, known as inducible caspase 9 (iCasp9), consists of a sequence from the human FK506-binding protein (modified FKBP12) with an F36V mutation linked to the gene encoding human caspase 9 (CASP9), which lacks its endogenous caspase activation and recruitment domain. FK506 (FKBP12-F36V) strongly binds to an inert small-molecule dimerizing agent, serving as a selectable marker. In the presence of this agent, the iCasp9 pro-molecule undergoes dimerization and activates the intrinsic apoptotic pathway, leading to the death of CAR T-cells expressing this fusion protein [186].

P-BCMA-101 (NCT03288493, NCT03741127) and P-BCMA-ALLO1 (NCT04960579) are autologous and allogeneic CAR-T therapies, respectively, that target BCMA with a second-generation CAR design (4-1BB-iCasp9), specifically incorporating this safety switch [36, 52, 104]. Additionally, a third-generation CAR-T targeting SLAMF-7 (CD28 or 4-1BB/CD3ζ-iCasp9) was investigated in a completed clinical trial (NCT03958656), and a fourth-generation CAR-T (CD28/CD27/CD3ζ-iCasp9) with multiple antigen targeting features is under evaluation in the clinical trial (NCT03125577). In instances of severe toxicity, the activation of inducible caspase 9 is initiated by administering Rimiducid as a safety switch activator [71] (Table 3).

##### EGFRt or CD20

One approach to eliminating toxic CAR T-cells is the selective targeting of these cells with antibodies [187]. This can be achieved by co-expressing cell surface proteins that are known to have established commercial mAbs against them, providing a suitable method for the specific reduction of CAR-Ts. This approach facilitates the selective elimination of CAR T-cells through mechanisms like antibody-dependent cell-mediated cytotoxicity (ADCC) and complement-dependent cytotoxicity (CDC) upon introducing a specific mAb [187, 188].

In MM research, the clinical application of mAbs like Cetuximab, which targets EGFRt, and Rituximab, designed for CD20, shows promise for their integration into CAR T-cell therapy [189]. Clinical trials have explored the use of second-generation CAR T-cells targeting BCMA (4-1BB-EGFRt) (NCT03070327, NCT03093168, NCT03502577), as well as third-generation CAR T-cells targeting both BCMA and CD19 (OX40/CD28-EGFRt) (NCT03455972), in MM patients. These trials focused on evaluating the efficacy of these CAR-T therapies, especially when administered alongside supplementary adjuvant treatments [71, 110, 190, 191]. Furthermore, it has been demonstrated that NKTR-255 therapy enhances ADCC with Rituximab and Cetuximab in patients who previously received CAR-T therapy [192]. However, the true efficacy of Cetuximab or Rituximab in MM patients remains a subject of ongoing investigation (Table 3).

#### Enhancing safety features of antibody binding domains in CAR T-cells

Multiple clinical trials have validated CAR T-cell therapies using human or humanized anti-BCMA scFv binding domains for R/R MM. These include trials for various products such as NCT03093168, NCT03070327, NCT03288493, NCT03741127, NCT04960579, NCT03338972, NCT04309981, NCT02546167, NCT04194931, NCT05201118, NCT03815383, NCT06045091, NCT04670055, NCT03975907, NCT03915184, NCT04003168 and NCT05698303. Additionally, ddBCMA, featuring a smaller-sized scFv consisting of 73 amino acids (NCT04155749), anti-BCMA VHH antigen binding domain (NCT03664661, NCT03661554), and anti-BCMA VH (engineered human antibody fragment, consisting of the variable domain of the heavy chain) (NCT03602612) have demonstrated the potential to decrease immunogenicity and achieve high response rates in patients with R/R MM [65, 193] (Table 3).

#### Supplementary treatments preceding or following CAR-T therapy in multiple myeloma

Commonly used PIs such as Bortezomib, Ixazomib, and Carfilzomib; IMiDs including Lenalidomide, Iberdomide, and Pomalidomide; and monoclonal antibodies such as anti-CD38 mAbs like Daratumumab, as well as anti-SLAMF7 mAbs like Elotuzumab, are often combined with chemotherapeutic drugs such as fludarabine and cyclophosphamide in MM CAR T-cell therapies [12, 194] (Table 4).

**Table 4:** Supplementary treatments preceding or following CAR-T therapy in multiple myeloma

						Adverse effect	Outcomes			
Target / CAR-T Name	Mechanism of Action	Combination. Regimen	Phase/ Study Name	Status	No. of Patient	Grade ≥ 3 CRS/ICANS,%	ORR,%	Median PFS, months	Location	NCT
BCMA (Cilta-cel)	Immunomodulator Proteasome inhibitor Anti-CD38 Ab Corticosteroid	Lenalidomide Bortezomib Daratumumab Dexamethasone	II (CARTITUDE-2)	Recruiting	169	0/0	95	Not yet reached	US	NCT04133636 [195]
BCMA (Cilta-cel)	Immunomodulator Proteasome inhibitor Anti-CD38 Ab Corticosteroid	Pomalidomide Bortezomib Daratumumab Dexamethasone	III (CARTITUDE-4)	Not recruiting	419	1/0	85	Not yet reached	US	NCT04181827 [79]
BCMA (Cilta-cel)	Immunomodulator Proteasome inhibitor Anti-CD38 Ab Corticosteroid Auto-Stem Cell Transplant	Lenalidomide Bortezomib Daratumumab Dexamethasone ASCT	III (CARTITUDE-6)	Recruiting	650	NA	NA	Not yet reached	US	NCT05257083
BCMA (Ide-cel)	Immunomodulator Auto-Stem Cell Transplant	Lenalidomide ASCT	Early II	Not recruiting	40	NA	NA	NA	US	NCT05032820
BCMA (Ide-cel)	Immunomodulator GPRC5D-directed BiTE Auto-Stem Cell Transplant	Lenalidomide Talquetamab ASCT	II (KarMMa-2)	Recruiting	264	2.7/0	83.8	11.4	US	NCT03601078 [196]
BCMA (Ide-cel)	Immunomodulator Immunomodulator Proteasome inhibitor Proteasome inhibitor Proteasome inhibitor Anti-CD38 mAb Anti-SLAMF7 mAb	Pomalidomide Lenalidomide Bortezomib Ixazomib Carfilzomib Daratumumab Elotuzumab	III (KarMMa-3)	Not recruiting	381	5/3	71	13.3	US	NCT03651128 [12]
BCMA (Ide-cel)	Immunomodulator Chemotherapeutic Chemotherapeutic	Lenalidomide Fludarabine Cyclophosphamide	I (KarMMa-4)	Completed	13	NA	NA	NA	US	NCT04196491
BCMA (Ide-cel)	Immunomodulator Immunomodulator Proteasome inhibitor Gamma secretase inhibitor Corticosteroid	Pomalidomide Iberdomide (CC-220) Bortezomib LY3039478(JSMD194) Dexamethasone	I/II (KarMMa-7)	Not recruiting	312	NA	NA	Not yet reached	US	NCT04855136
BCMA (Ide-cel)	Immunomodulator Chemotherapeutic Chemotherapeutic	Lenalidomide Fludarabine Cyclophosphamide	III (KarMMa-9)	Recruiting	618	NA	NA	Not yet reached	US	NCT06045806
BCMA (ALLO-715)	Gamma secretase inhibitor anti-CD52 Ab	Nirogacestat ALLO-647	I (UNIVERSAL)	Not recruiting	132	2/0	61.5	Not yet reached	US	NCT04093596 [38]
BCMA (ALLO-605)	anti-CD52 Ab	ALLO-647	I/II (IGNITE)	Not recruiting	136	NA	NA	NA	US	NCT05000450
BCMA (PBCAR269A)	Gamma secretase inhibitor	Nirogacestat	I	Terminated	48	NA	NA	NA	US	NCT04171843
BCMA	Gamma secretase inhibitor	LY3039478 (JSMD194)	Early I	Terminated	18	NA	NA	NA	US	NCT03502577 [110]
BCMA	Immunomodulator Antibiotic Corticosteroid	Lenalidomide Clarithromycin Dexamethasone	III	Recruiting	20	NA	NA	NA	China	NCT04287660
BCMA (CT103A)	selective inhibitor of nuclear transport	Selinexor	I	Unknown	20	NA	NA	Not yet reached	China	NCT05201118
BCMA	GPRC5D-directed BiTE	Talquetamab	II	Recruiting	17	NA	NA	NA	US	NCT06066346
BCMA	BCMA-targeted ADC	Belantamab Mafodotin	II (EMBRACE)	Recruiting	45	NA	NA	NA	US	NCT05117008
BCMA	BCMA-targeted ADC Immunomodulator Proteasome inhibitor Corticosteroid	Belantamab Mafodotin Pomalidomide Carfilzomib Dexamethasone	II	Recruiting	83	NA	NA	NA	US	NCT05789303
BCMA (C-CAR088)	Auto-Stem Cell Transplant	ASCT	I/II	Recruiting	20	0/0	100	Not yet reached	China	NCT05632380 [197]
BCMA	Auto-Stem Cell Transplant	ASCT	I	Recruiting	20	NA	NA	NA	US	NCT05887167
CD19/BCMA	Auto-Stem Cell Transplant	ASCT	I/II	Recruiting	15	0/0	100	NA	China	NCT03455972 [82]
BCMA (MCARH171)	Immunomodulator Radiation Therapy	Lenalidomide	I	Not recruiting	20	2/0	64	Not yet reached	US	NCT03070327 [159]
BCMA	Radiation Therapy		II	Recruiting	30	NA	NA	NA	US	NCT05336383
BCMA (UPenn)	Radiation Therapy		I	Completed	25	32/12	48	2.7	US	NCT02546167 [72]
BCMA	CAR T re-treatment		I	Unknown	20	NA	NA	NA	China	NCT03672253

B-Cell Maturation Antigen (BCMA), Cluster of Differentiation (CD), Bispecific T-cell engagers (BiTEs), Antibody–drug conjugate (ADC), Auto-Stem Cell Transplant (ASCT), Cytokine release syndrome (CRS), Immune effector cell-associated neurotoxicity syndrome (ICANS), Overall response rate (ORR), and Progression-free survival (PFS)

Isatuximab, another anti-CD38 mAb approved for adult patients with R/R MM, can potentially enhance CAR-T efficacy [71]. However, the decline in CD38 antigen presence and the emergence of CD38-negative plasma cells pose a significant challenge in R/R MM clinical trials. Research findings suggest that all-trans retinoic acid (ATRA) can enhance CD38 expression, leading to improved efficacy of CD38 monoclonal antibodies and anti-CD38 CAR T-cell therapy [198]. This could expand the repertoire of treatment options available for multiple myeloma.

Mounting evidence underscores the transformative role of various immunotherapies, such as novel monoclonal antibodies, bispecific T-cell engagers (BiTEs), and antibody–drug conjugate (ADC) therapies as innovative treatment modalities for R/R MM [199]. These approaches can potentially complement CAR T-cell regimens. Clinical trials are evaluating several BiTEs targeting BCMA, GPRC5D, and FcRH5 in heavily pretreated MM patients. BCMA-targeted BiTEs like Teclistamab and Elranatamab, GPRC5D-directed BiTE Talquetamab, and BCMA-targeted ADC Belantamab mafodotin have already gained approval for R/R MM [200–203]. Clinical trials are currently assessing the effectiveness of Belantamab mafodotin (NCT05117008, NCT05789303) and Talquetamab (NCT06066346) within the context of MM CAR T-cell therapies (Table 4). Several BiTEs are currently in clinical trials for MM treatment, showing promise for FDA approval and potential integration with CAR T-cell therapies. Linvoseltamab and F182112 target BCMA, while Cevostamab targets FcRH5. [204]. Early results look promising, with Linvoseltamab showing a 64% response rate and F182112 showing a 43.8% response rate [205, 206].

In a study focusing on a fully human BCMA-targeting CAR T-cell therapy (CT103A), researchers have incorporated Selinexor, a selective inhibitor of nuclear export. Selinexor functions by disrupting the activity of exportin-1 (XPO1), a protein crucial for transporting various proteins and ribonucleoproteins from the nucleus to the cytoplasm. This inhibition prevents the nuclear export of tumor suppressor proteins and cell cycle regulators, leading to their accumulation in the nucleus [207, 208]. The potential of Selinexor in combination with CAR T-cell treatment strategies is being investigated for managing R/R MM (NCT05201118) (Table 4).

sBuilding on this approach, additional research studies are currently underway to further enhance the treatment of multiple myeloma patients. These studies involve various strategies, including autologous hematopoietic stem cell (Auto-HSC) transplantation followed by CART-19/BCMA infusion (NCT03455972) or in combination with CAR T-BCMA therapy (NCT05887167, NCT05632380, NCT05257083), radiation therapy post CAR T-BCMA therapy (NCT05336383, NCT03070327, NCT02546167) [209–211], and CAR T-cell re-treatment following previous CAR T-cell therapy (NCT03672253) [65] (Table 4).

## Perspectives on the future of MM-CAR T cells in clinical trials

For most MM patients with several previous treatments, allogeneic leukapheresis products from healthy donors offer more cost-effective and life-preserving opportunities [53]. Despite allogeneic CAR T-cells’ short in vivo lifespan, patient outcomes have been encouraging, necessitating further research [51]. Following the FDA’s grant of fast-track designation to ALLO-605 (NCT05000450) in June 2021 and its inclusion in the FDA orphan product development program in April 2022, the decision to advance from Phase II to Phase III is expected to be guided by insights from global data concerning the success rate of transitioning between these clinical trial phases.

Furthermore, MM’s autologous CAR T-cell therapy field has seen significant advancements, with several notable achievements. BCMA CAR T-cell therapy NXC-201 (formerly HBI0101) (NCT04720313) demonstrated a 90% ORR in relapsed/refractory MM patients and a 100% ORR in heavily pre-treated relapsed/refractory amyloid light chain (AL) amyloidosis in the phase Ia/Ib NEXICART-1 trial [212, 213]. Additionally, BCMA/CD19 CAR T-cell (GC012F) trials (NCT04236011, NCT04182581, NCT04617704, NCT04935580, NCT05850234, ChiCTR2100047061) achieved a higher 90% ORR [32, 48, 49, 214]. The autologous GPRC5D CAR T-cell therapy OriCAR-017 showed nearly a 100% ORR in its phase I POLARIS trial (NCT05016778) [87, 215]. These promising results have led to orphan drug designations from the FDA for MM treatment.

## Conclusion

Although initial clinical trials demonstrated noteworthy potential for traditional CAR T-cells in the treatment of multiple myeloma, the limited accessibility or persistence, considerable costs, and time involved in manufacturing, coupled with challenges such as patient relapse or side effects following anti-BCMA CAR-T therapy, emphasize the necessity for enhanced efficacy. This situation presents a complex and demanding challenge in the field. Consequently, clinical and basic science have taken on a crucial role in three key objectives: enhancing CAR T-cell manufacturing and persistence, developing insights into disease features, and addressing supplementary therapeutic aspects both prior to and following CAR T-cell therapy in the context of MM.

In this article, we begin by examining strategies employed to enhance the persistence of CAR T-cells through structural and administration optimization. This entails the consideration of immunophenotype characterization factors, including achieving a specific CD4/CD8 ratio, utilizing CD8 + or T stem cell memory phenotype, enhancing CAR T-cell cultures, or equipping them with pro-inflammatory cytokines or ligands to promote a memory CAR T-cell-enriched phenotype [28, 36, 42, 43]. Additionally, it involves the exploration of rapid CAR-T manufacturing methods through novel genetic modifications to reduce transplant timing and costs while maintaining lower-differentiated T cell phenotypes [47, 48]. Initiating CAR T-cell therapy at earlier stages of treatment and the development of universal allogeneic CAR T-cells or even NK-CAR cells introduce new opportunities for patients with high-risk or relapsed/refractory multiple myeloma (R/R MM) [51–53, 64] (Table 1).

Regarding the second aspect, the article delves into the intricacies associated with the inherent factors of MM. These complexities include the heterogeneous nature of the cancer and the potential for BCMA evasion or shedding, which can be tackled through innovative CAR designs, such as dual or multitarget CAR T-cells [71, 104] (Table 2). The use of gamma-secretase inhibitors has also been explored to address these challenges [70, 105, 110] (Table 4). Moreover, strategies to counteract the presence of an immunosuppressive tumor microenvironment have been discussed. This involves inhibiting intracellular exhaustion signals through genetic modifications or employing inhibitors as part of the treatment [135, 137, 138, 141] (Table 2).

The final category encompasses clinical aspects associated with CAR T-cell transplantation. This involves addressing unwanted side effects that may occur after CAR T-cell transplantation, such as cytokine release syndrome, neurotoxicity, or cytopenia. These side effects can be managed by utilizing pharmacological immunosuppressive agents in combination or suicide genes that induce direct interactions leading to CAR T-cell death through spatial drugs. Further, the immunogenicity of CAR T-cells can be mitigated by designing humanized scFv or VHH for their recognition sites [172, 216] (Table 3). It also includes the incorporation of additional treatments like proteasome inhibitors, immunomodulators, monoclonal antibodies, bispecific T-cell engagers, antibody–drug conjugates, chemotherapy, corticosteroid therapy, radiation therapy, and autologous hematopoietic stem cell transplantation in combination with CAR-T therapy to enhance its efficacy [66, 194, 201, 202] (Table 4).

## Data Availability

Not applicable.

## Abbreviations

MM: Multiple myeloma
CAR: Chimeric antigen receptor
BCMA: B-cell maturation antigen
IMiD: Immunomodulatory drugs
PI: Proteasome inhibitor
mAb: Monoclonal antibody
PCs: Plasma cells
BM: Bone marrow
R/R MM: Relapsed/ refractory multiple myeloma
CRS: Cytokine release syndrome
ICANS: Immune effector cell-associated neurotoxicity syndrome
TME: Tumor microenvironment
ALL: Acute Lymphoblastic Leukemia
DLBCL: Diffuse Large B cell Lymphoma
FL: Follicular lymphoma
ScFv: Single-chain variable fragment
MHC-I: Major histocompatibility complex class I
MIC: MHC class I chain-related protein
NKG2D: Natural-killer group 2 member D
GPRC5D: G protein-coupled receptor class C group 5 member D
BAFF: B-cell activating factor
APRIL: A proliferation-inducing ligand
TRIPRIL: Trimeric APRIL-based CAR
CAF: Cancer-associated fibroblast
SLAMF-7: Signaling lymphocyte activation molecule family-7
FAP: Fibroblast activation protein
ULBP: Unique long 16-binding protein
TnMUC1: Tn antigen on membrane mucin 1
c-Met: Cellular-mesenchymal-epithelial transition
NY-ESO-1: New York Esophageal Squamous Cell Carcinoma-1
ddBCMA: D-domain-based antigen binder B-Cell Maturation Antigen
MSLN: Mesothelin
NK: Natural Killer
CB-NK: Cord blood-derived natural killer
IPSCs: Induced Pluripotent Stem Cells
MDSC: Myeloid-derived suppressor cells
GM-CSF: Granulocyte–macrophage colony stimulating factor
PD-1: Programmed death protein 1
CTLA4: Cytotoxic T-lymphocyte associated protein 4
LAG3: Lymphocyte-activation gene 3
TIM-3: T-cell immunoglobulin mucin-3
BTLA: B and T-lymphocyte attenuator
TGF-β: Transforming Growth Factor-beta
VEGF: Vascular endothelial growth factor
PGE2: Prostaglandin E2
TNF-α: Tumor Necrosis Factor-alpha
IFN-γ: Interferon-gamma
TIGIT: T-cell immunoreceptor with Ig and ITIM domains
GSI: Gamma secretase inhibitor
TLS: Tumor lysis syndrome
GvHD: Graft-versus-host disease
PI3K: Phosphoinositide 3-kinase
MAS: Macrophage activation syndrome
HLH: Hemophagocytic lymphohistiocytosis
IL-6RA: IL-6 receptor antagonist
IL-1RA: IL-1 receptor antagonist
HSV-TK: Herpes simplex virus thymidine kinase
tEGFR/EGFRt: Truncated epidermal growth factor receptor
LCK: Lymphocyte-specific protein tyrosine kinase
GCV: Ganciclovir
FADD: Fas-associated death domain protein
iCasp9: Inducible caspase 9
ADCC: Antibody-dependent cell-mediated cytotoxicity
CDC: Complement-dependent cytotoxicity
MDS: Myelodysplastic syndrome
SAMT: Subsequent anti-myeloma therapy
BiTE: Bispecific T-cell engager
ADC: Antibody–drug conjugate

## Data availability

No datasets were generated or analysed during the current study.
